# Supplementary material for: Qualitative documentary analysis of guidance on information provision and consent for the introduction of innovative invasive procedures including surgeries within NHS organisations’ policies in England and Wales
Source: BMJ Open. 2022 Sep 1;12(9):e059228. doi: 10.1136/bmjopen-2021-059228 (PMC9438078; doi:10.1136/bmjopen-2021-059228)
Supplement: Supplementary data [file bmjopen-2021-059228supp001.pdf]

Supplementary file

Appendix 1 information to tell patients about new IP/Ds

Policy number	Newness or special status (N=73, 80%)	Referenced PIL or written information (N=68, 75%)	Alternative treatments (N=61, 67%)	Uncertainty regarding procedure (N=57, 63%)	Surgeon's experience (N=57, 63%)	Mandated PIL (N=30, 33%)	Total
14	Yes	Yes	Yes	Yes	Yes	Yes	6/6
19	Yes	Yes	Yes	Yes	Yes	Yes	6/6
22	Yes	Yes	Yes	Yes	Yes	Yes	6/6
41	Yes	Yes	Yes	Yes	Yes	Yes	6/6
49	Yes	Yes	Yes	Yes	Yes	Yes	6/6
51	Yes	Yes	Yes	Yes	Yes	Yes	6/6
55	Yes	Yes	Yes	Yes	Yes	Yes	6/6
58	Yes	Yes	Yes	Yes	Yes	Yes	6/6
81	Yes	Yes	Yes	Yes	Yes	Yes	6/6
88	Yes	Yes	Yes	Yes	Yes	Yes	6/6
95	Yes	Yes	Yes	Yes	Yes	Yes	6/6
146	Yes	Yes	Yes	Yes	Yes	Yes	6/6
91	Yes	Yes	Yes	Yes	Yes	Yes	6/6
13	Yes	Yes	Yes	Yes	No	Yes	5/6
21	Yes	Yes	No	Yes	Yes	Yes	5/6
26	Yes	Yes	Yes	Yes	Yes	No	5/6
38	Yes	Yes	Yes	Yes	Yes	No	5/6
39	Yes	Yes	Yes	No	Yes	Yes	5/6
50	Yes	Yes	Yes	Yes	No	Yes	5/6
54	Yes	Yes	Yes	Yes	Yes	No	5/6
56	Yes	Yes	Yes	Yes	Yes	No	5/6
65	Yes	Yes	No	Yes	Yes	Yes	5/6
76	Yes	Yes	Yes	Yes	Yes	No	5/6
105	Yes	Yes	Yes	Yes	Yes	No	5/6

110	Yes	Yes	Yes	Yes	Yes	No	5/6
118	Yes	Yes	Yes	Yes	Yes	No	5/6
122	Yes	Yes	Yes	Yes	Yes	No	5/6
126	Yes	Yes	Yes	Yes	Yes	No	5/6
132	Yes	Yes	Yes	Yes	Yes	No	5/6
150	Yes	Yes	Yes	Yes	Yes	No	5/6
153	Yes	Yes	Yes	Yes	Yes	No	5/6
12	Yes	Yes	Yes	yes	Yes	No	5/6
3	No	Yes	Yes	Yes	No	Yes	4/6
4	Yes	Yes	Yes	Yes	No	No	4/6
7	Yes	Yes	No	No	Yes	Yes	4/6
10	Yes	No	Yes	Yes	Yes	No	4/6
18	Yes	No	Yes	Yes	Yes	No	4/6
32	Yes	No	Yes	Yes	Yes	No	4/6
42	Yes	No	Yes	Yes	Yes	No	4/6
43	Yes	Yes	No	Yes	Yes	No	4/6
48	Yes	No	Yes	Yes	Yes	No	4/6
57	Yes	Yes	Yes	No	No	Yes	4/6
63	Yes	No	Yes	Yes	Yes	No	4/6
71	Yes	No	Yes	Yes	Yes	No	4/6
82	Yes	No	Yes	Yes	Yes	No	4/6
84	Yes	Yes	Yes	Yes	No	No	4/6
92	Yes	Yes	Yes	Yes	No	No	4/6
104	Yes	No	Yes	Yes	Yes	No	4/6
107	Yes	No	Yes	Yes	Yes	No	4/6
113	Yes	No	Yes	Yes	Yes	No	4/6
125	Yes	No	Yes	Yes	Yes	No	4/6
127	Yes	No	Yes	Yes	Yes	No	4/6
128	Yes	Yes	No	Yes	Yes	No	4/6
138	Yes	Yes	Yes	Yes	No	No	4/6

2	No	Yes	No	Yes	No	Yes	3/6
15	Yes	Yes	No	No	Yes	No	3/6
25	Yes	Yes	Yes	No	No	No	3/6
33	Yes	Yes	Yes	No	No	No	3/6
37	Yes	Yes	No	No	Yes	No	3/6
53	Yes	No	Yes	Yes	No	No	3/6
83	Yes	No	Yes	Yes	No	No	3/6
100	Yes	Yes	No	No	No	Yes	3/6
121	Yes	Yes	No	No	Yes	No	3/6
123	Yes	No	Yes	Yes	No	No	3/6
124	Yes	No	Yes	Yes	No	No	3/6
130	Yes	Yes	No	No	Yes	No	3/6
137	Yes	Yes	No	No	No	Yes	3/6
148	No	Yes	Yes	No	Yes	No	3/6
154	No	Yes	No	No	Yes	Yes	3/6
156	Yes	Yes	No	No	No	Yes	3/6
1	Yes	No	No	No	Yes	No	2/6
23	No	Yes	No	No	No	Yes	2/6
30	No	Yes	No	No	No	Yes	2/6
36	Yes	No	Yes	No	No	No	2/6
52	No	Yes	No	No	No	Yes	2/6
59	Yes	No	No	No	Yes	No	2/6
60	Yes	Yes	No	No	No	No	2/6
64	No	Yes	No	No	Yes	No	2/6
70	Yes	No	No	No	Yes	No	2/6
80	Yes	No	No	Yes	No	No	2/6
94	No	No	Yes	Yes	No	No	2/6
96	No	Yes	Yes	No	No	No	2/6
102	No	Yes	Yes	No	No	No	2/6
131	Yes	Yes	No	No	No	No	2/6

134	No	No	Yes	No	Yes	No	2/6
147	No	Yes	No	No	No	Yes	2/6
34	No	Yes	No	No	No	No	1/6
61	No	Yes	No	No	No	No	1/6
68	No	Yes	No	No	No	No	1/6
109	No	Yes	No	No	No	No	1/6
139	No	Yes	No	No	No	No	1/6

Appendix 2. Recommendation for information to be included in written patient information sheets

Policy Number	cc	Alternative Treatments (N=5, 26%)	NICE Status (N=4, 21%)	Special status/newness (N=9, 47%)	Surgeon's Experience (N=3, 16%)	Evidence (N=1, 5%)	Total
52	Yes	Yes	Yes	No	No	No	3/6
55	No	No	No	Yes	Yes	Yes	3/6
102	Yes	Yes	Yes	No	No	No	3/6
3	Yes	Yes	No	No	No	No	2/6
12	No	No	Yes	Yes	No	No	2/6
41	Yes	No	No	Yes	No	No	2/6
49	Yes	No	No	Yes	No	No	2/6
51	No	No	No	Yes	Yes	No	2/6
88	No	No	No	Yes	Yes	No	2/6
92	Yes	Yes	No	No	No	No	2/6
137	Yes	No	No	Yes	No	No	2/6
13	No	No	No	Yes	No	No	1/6
25	No	No	No	Yes	No	No	1/6
30	No	No	Yes	No	No	No	1/6
50	Yes	No	No	No	No	No	1/6
65	No	Yes	No	No	No	No	1/6

109	Yes	No	No	No	No	No	1/6
147	Yes	No	No	No	No	No	1/6
156	Yes	No	No	No	No	No	1/6
