# Supplementary material for: Qualitative documentary analysis of guidance on information provision and consent for the introduction of innovative invasive procedures including surgeries within NHS organisations’ policies in England and Wales
Source: BMJ Open. 2022 Sep 1;12(9):e059228. doi: 10.1136/bmjopen-2021-059228 (PMC9438078; doi:10.1136/bmjopen-2021-059228)
Supplement: Author's manuscript [file bmjopen-2021-059228.draft_revisions.pdf]

BMJ Open

BMJ Open is committed to open peer review. As part of this commitment we make the peer review history of every article we publish publicly available.

When an article is published we post the peer reviewers' comments and the authors' responses online. We also post the versions of the paper that were used during peer review. These are the versions that the peer review comments apply to.

The versions of the paper that follow are the versions that were submitted during the peer review process. They are not the versions of record or the final published versions. They should not be cited or distributed as the published version of this manuscript.

BMJ Open is an open access journal and the full, final, typeset and author-corrected version of record of the manuscript is available on our site with no access controls, subscription charges or pay-per-view fees (http://bmjopen.bmj.com).

If you have any questions on BMJ Open's open peer review process please email info.bmjopen@bmj.com

BMJ Open

A review of guidance on information provision and consent for the introduction of innovative invasive procedures within NHS organisation policies in England and Wales.

Journal:	*BMJ Open*
Manuscript ID	bmjopen-2021-059228
Article Type:	Original research
Date Submitted by the Author:	30-Nov-2021

Complete List of Authors:	Ochieng, Cynthia; University of Bristol, Population Health Sciences<br>Richards, Hollie; University of Bristol, Population Health Sciences<br>Zahra, Jesmond; University of Bristol, Population Health Sciences<br>Cousins, Sian; University of Bristol, Population Health Sciences<br>Elliott, Daisy; University of Bristol, Population Health Sciences<br>Wilson, Nicholas; University of Bristol, Population Health Sciences<br>Paramasivan, Sangeetha ; University of Bristol, Population Health Sciences<br>Avery, Kerry; University of Bristol, Population Health Sciences<br>Mathews, Johnny; University of Bristol, Population Health Sciences<br>Main, Barry; University of Bristol, Population Health Sciences; University Hospitals Bristol NHS Foundation Trust<br>Hinchliffe, Robert; University of Bristol, Population Health Sciences; North Bristol NHS Trust<br>Blencowe, Natalie; University of Bristol, Population Health Sciences; University Hospitals Bristol NHS Foundation Trust<br>Blazeby, Jane; University of Bristol, Population Health Sciences; University Hospitals Bristol NHS Foundation Trust

Keywords:	Health policy < HEALTH SERVICES ADMINISTRATION & MANAGEMENT, Clinical governance < HEALTH SERVICES ADMINISTRATION & MANAGEMENT, ETHICS (see Medical Ethics), Protocols & guidelines < HEALTH SERVICES ADMINISTRATION & MANAGEMENT, SURGERY

SCHOLARONE™
Manuscripts

I, the Submitting Author has the right to grant and does grant on behalf of all authors of the Work (as defined in the below author licence), an exclusive licence and/or a non-exclusive licence for contributions from authors who are: i) UK Crown employees; ii) where BMJ has agreed a CC-BY licence shall apply, and/or iii) in accordance with the terms applicable for US Federal Government officers or employees acting as part of their official duties; on a worldwide, perpetual, irrevocable, royalty-free basis to BMJ Publishing Group Ltd ("BMJ") its licensees and where the relevant Journal is co-owned by BMJ to the co-owners of the Journal, to publish the Work in this journal and any other BMJ products and to exploit all rights, as set out in our licence.

The Submitting Author accepts and understands that any supply made under these terms is made by BMJ to the Submitting Author unless you are acting as an employee on behalf of your employer or a postgraduate student of an affiliated institution which is paying any applicable article publishing charge ("APC") for Open Access articles. Where the Submitting Author wishes to make the Work available on an Open Access basis (and intends to pay the relevant APC), the terms of reuse of such Open Access shall be governed by a Creative Commons licence – details of these licences and which Creative Commons licence will apply to this Work are set out in our licence referred to above.

Other than as permitted in any relevant BMJ Author's Self Archiving Policies, I confirm this Work has not been accepted for publication elsewhere, is not being considered for publication elsewhere and does not duplicate material already published. I confirm all authors consent to publication of this Work and authorise the granting of this licence.

For peer review only

A review of guidance on information provision and consent for the introduction of innovative invasive procedures within NHS organisation policies in England and Wales.

Submission category: Original research

Authors: Cynthia A Ochieng (0000-0002-5574-6059), senior research associate, c.ochieng@bristol.ac.uk[1], Hollie Richards (0000-0001-9181-7005), senior research associate, hollie.richards@bristol.ac.uk[1], Jesmond Zahra (0000-0002-7947-2216), senior research associate, jesmond.zahra@defra.gov.uk[1] , Sian Cousins (0000-0003-0088-841X), research fellow, sian.cousins@bristol.ac.uk[1], Daisy Elliott (0000-0001-8143-9549), research fellow, daisy.elliott@bristol.ac.uk[1], Nicholas Wilson, senior research associate, nicholas.wilson@bristol.ac.uk[1], Sangeetha Paramasivan (0000-0001-7329-9574), research fellow, sangeetha.paramasivan@bristol.ac.uk[2], Kerry NL Avery (0000-0001-5477-2418), senior lecturer, kerry.avery@bristol.ac.uk[1], Johnny Mathews, PhD fellow, johnny.mathews@bristol.ac.uk[1], Barry G Main (0000-0003-0622-805X), consultant senior lecturer, b.g.main@bristol.ac.uk[1,3], Robert Hinchliffe (0000-0002-6370-0800), clinical professor of vascular surgery, robert.hinchliffe@bristol.ac.uk[1,4], Natalie S Blencowe (0000-0002-6111-2175), associate professor in surgery, natalie.blencowe@bristol.ac.uk[1,3], Jane M Blazeby (0000-0002-3354-3330), professor of surgery, j.m.blazeby@bristol.ac.uk[1,3],

[1] National Institute for Health Research Bristol and Weston Biomedical Research Centre, Surgical Innovation Theme, Centre for Surgical Research, Population Health Sciences, Bristol Medical School, University of Bristol, 39 Whatley Road, Bristol, BS8 2PS

[2]Population Health Sciences, Bristol Medical School, University of Bristol, 39 Whatley Road, Bristol, BS8 2PS

[3]University Hospitals Bristol NHS Foundation Trust, Bristol, UK. BS2 8HW

[4]North Bristol NHS Trust, Bristol, United Kingdom

Correspondence to: Cynthia A Ochieng c.ochieng@bristol.ac.uk

Word count: 3212 (excluding title page, abstract, tables and references)

Abstract

Objective- To review guidance, included in written local NHS organisation policies, on information provision and consent for the introduction of new invasive procedures and devices (IP/Ds).

Design- Content analysis and thematic synthesis of data on patient information provision and consent extracted from policies for the introduction of IP/Ds from NHS organisations in England and Wales.

Setting- NHS trusts in England and health boards in Wales, United Kingdom.

Participants- Between December 2017 and July 2018 150 acute trusts in England and seven health boards in Wales were approached for their policies for the introduction of new IP/Ds. 123 policies were received, 11 did not fit the inclusion criteria and a further policy was included from a trust website resulting in 113 policies included for review.

Results- From the 113 policies, 22 did not include any statements on informed consent/information provision or lacked guidance on the information to be provided to patients and were hence excluded. Consequently, 91 written local NHS policies were included in the final dataset. Within the guidance obtained, variation existed on disclosure of the procedure's novelty, potential risks, benefits, uncertainties, alternative treatments, and surgeon's experience. Few policies stated that clinicians should discuss the existing evidence associated with a procedure. Additionally, whilst the majority of policies referred to patients needing written information, this was often not mandated and few policies specified the information to be included.

Conclusions- Nearly a fifth of all the policies lacked guidance on information to be provided to patients. There was variability in the policy documents regarding what patients should be told about innovative procedures. Further research is needed to ascertain the information and level of detail appropriate for patients when considering innovative procedures. A core information set including patients' and clinicians' views is required to address variability around information provision/ consent for innovative procedures.

For peer review only - http://bmjopen.bmj.com/site/about/guidelines.xhtml

Strengths and limitations of this study

- This is the first study to systematically review guidance within NHS policies on patient information provision and consent when introducing new invasive procedures and devices in England and Wales.
- The study explores not just the types of information included in the policies but also the level of detail.
- The study cannot ascertain if and how informed consent practices are implemented by clinicians and/or whether other regulatory processes play a role in patient consent.

For peer review only

For peer review only - http://bmjopen.bmj.com/site/about/guidelines.xhtml

Introduction

Innovation in surgery has transformed how clinical care is delivered; new surgical techniques and technologies have resulted in improved outcomes, shorter recovery times and fewer adverse events following surgery.[1-3] Nonetheless, as documented in recent reports, surgical innovations are sometimes introduced into clinical practice without sufficient evaluation and pose unknown risks to patients.[4 5] Introducing new invasive procedures via research pathways helps ensure clinicians comply with legal, ethical and scientific standards.[6] Despite increasing demand for evidence-based practice, new surgical procedures are commonly delivered prior to appropriate evaluation and without research ethics approval.[7-11] Working in conjunction with the National Institute for Health and Care Excellence (NICE), the UK Department of Health and Social Care (DHSC) published guidelines for managing the introduction of new invasive procedures within the NHS.[12 13] These are relevant to NHS local 'new procedures committees/clinical effectiveness committees'.[14] These guidelines included sections on information provision and obtaining consent from patients particularly for procedures introduced outside the remit of research protocols; these were distributed to NHS chief executives via the Health Service Circular (HSC) in 2003.[12]

Patient autonomy is considered a cornerstone of modern healthcare provision.[15] Clinicians must work in partnership with patients and take their views, goals and circumstances into consideration when establishing best treatment.[16 17] There is a scarcity of empirical research exploring informed consent in the context of surgical innovation, consequently, little is known about how innovative surgeries are, or should be, discussed with patients[18]. When innovations are introduced as part of a research study, ethical review processes help ensure that patient information is appropriate and includes detail of the potential risks and benefits.[19] However, when procedures are introduced outside the remit of research studies these safeguards regarding patient consent for innovative surgeries are not always adhered to.[20] While guidelines on consent practices for new technologies have recently been published,[2] there persists a paucity of research investigating how these are implemented in clinical practice. According to the 2003 recommendations, approvals for the delivery of new invasive procedures, which have not been evaluated or recommended by NICE, should only be granted if special consent measures are put in place.[12]

For peer review only - http://bmjopen.bmj.com/site/about/guidelines.xhtml

Most individual NHS organisations have policies in place regarding the introduction of new invasive procedures and devices (IP/D),[14 21 22] however, it is unknown what guidance these policies provide about information provision to patients undergoing innovative procedures. This review aimed to investigate information provision and consent measures for the introduction of new invasive procedures and devices described in policy documents within NHS organisations in England (trusts) and Wales (health boards).

Methods

Study design

A comprehensive review of guidance on information provision and consent within NHS organisations' policies for the introduction of IP/Ds was undertaken. An innovative IP/D was defined as any procedure where access is gained via an incision, natural orifice or percutaneous puncture or involving devices used inside the body[23]. The findings reported here form part of the INTRODUCE Study.[14]

Data collection

Between December 2017 and July 2018 all acute trusts in England (n=150) and health boards in Wales (n=7) were contacted (via email and telephone calls) and requested for their policies relating to the introduction of an innovative P/D.[14] Of these, 147 responded and 127 stated that they had the requested policies. Of the 127 trusts and health boards, four did not share their policies with the research team. Consequently, 123 policies were received of which 11 were excluded from the review for: being device management policies (n=6), application forms only (n=3), committee terms of reference (n=1), and local safety standards for invasive procedures (n=1). Further detail on the eligibility criteria have been published in the main protocol.[14] In addition, one policy was obtained online from the trust website, resulting in 113 policies available for review.

Data extraction and analysis

For peer review only - http://bmjopen.bmj.com/site/about/guidelines.xhtml

Policies were initially read and re-read by HSR. All text relating to new procedures and devices including information provision and consent practice, was extracted verbatim by HSR into a data extraction form. The data extraction form was iteratively developed through reviewing policy documents and previous literature[14] and stored on the online surveys portal (formerly Bristol online surveys). 20% of the policies were independently reviewed and data extraction was conducted by SC, JMB, JM, HR and JZ. Any disagreements among reviewers were discussed and resolved.

Extracted data relating to information provision and consent were then imported into NVivo (version 12) by JZ for review and analysis using methods derived from content analysis[24] and thematic synthesis.[25 26] Text was initially read and re-read to ensure familiarisation with the data. Following this, coding categories were inductively identified through reviewing data and discussions within the team. In total, five coding categories were included in the final thematic framework. To ensure this framework fully encapsulated the data, it was piloted on a sample of policies (15%), by two analysts (JZ and JM) before being independently applied to the full dataset. Data within each theme were reviewed to investigate variations between policy statements on consent.

Patient and public involvement

The review was conducted under the National Institute for Health Research (NIHR) Bristol & Weston Biomedical Research Centre (BRC) Surgical Innovation theme, which conducts research aimed at improving safety and transparent translation of innovative procedures and/or devices to clinical practice. The NIHR Bristol BRC has a dedicated patient and public involvement (PPI) group, where previous surgical patients contribute their views on different projects. In this study, PPI contributors included their views and experiences of the information they received or would have wanted to receive before and after having innovative surgeries. The PPI group were supportive of this study. Our PPI group agreed with the methods applied for the review of the policy documents as well as the resultant findings.

Results

For peer review only - http://bmjopen.bmj.com/site/about/guidelines.xhtml

Policies included

Of the 113 policies reviewed, seven did not include any statements on informed consent/information provision. Of the remaining 106, 15 provided only brief statements on informed consent (e.g. “consent must be obtained”) and did not provide any guidance on the content of information to be provided to patients. This highlights that 19% (22/113) of all the policies did not include any statement on informed consent or lacked guidance on information to be provided to patients. Following discussions within the team, these 15 policies were excluded from further analysis. In total, text from 91 local NHS policies was included in the final dataset (see figure 1- flowchart of policy documents reviewed).

Findings

Five categories were included in the final thematic framework and grouped into two main themes: statements about the information clinicians should disclose about a new IP/D being delivered with local NHS policy approval, and guidance on the use of written information for this situation. The findings in each of the five categories from the analytic framework are described in detail below. Very few policies included guidance on all of the five categories identified (n = 14, see appendix 1).

Statements specifying what information clinicians should disclose prior to delivering an IP/D

“New/special” status of an IP/D

Of the final 91 policies reviewed, 73 (80%) policies stipulated that clinicians should discuss the novelty of a procedure with patients, however, the terminology used within policies varied. 48 (53%) stated the “special status” (n = 44) or “status” (n = 4) of a procedure should be disclosed. This terminology mirrored that used in the HSC document distributed in 2003[12]:

> *“All patients offered the procedure are made aware of the special status of the procedure”*

For peer review only - http://bmjopen.bmj.com/site/about/guidelines.xhtml

Of the remaining policies, 20 (22%) used the term "new" and five (5%) included statements that used both "new" and "special status". Notably, only one policy stated clinicians should explain whether the procedure was "new to the trust" or "completely new":

> *"An explanation must be given to the patient as to whether the new technique / procedure is either new to the Trust but established elsewhere, or, is a completely new technique / procedure." Policy 81*

Three (3%) policies provided guidance on how long clinicians should consider a procedure as new and introduce it to patients as such, however, guidance varied between policies. Specific recommendations were that, i) standard consent processes should be applied following a successful audit of the procedure, ii) clinicians should review their results and decide when a procedure is an established part of practice, and iii) procedures should be introduced as "new" to the first five patients.

Uncertainty, evidence, risks and benefits of a procedure

58 (64%) policies stated that clinicians should discuss uncertainties surrounding safety and efficacy, and/or risks and benefits. Of these, 40 (44%) included an extract from the HSC document[12]:

> *"Patients need to understand that the procedure's safety and efficacy is uncertain and be informed about the anticipated benefits and possible adverse effects of the procedure"*

12 (13%) of the remaining policies simply stated that patients need to be "*informed*" that some uncertainty existed for a procedure.

Five policies provided more comprehensive guidelines for clinicians to follow when discussing uncertainty. For example, one policy stated that the frequency of procedure complications should also be disclosed:

> *"The most important consideration is that patients (or their parents or carers, when appropriate) should be informed and should understand the risks when offered the procedure. This always means telling them the known risks, but in addition it may mean telling them that there is*

For peer review only - http://bmjopen.bmj.com/site/about/guidelines.xhtml

uncertainty about the frequency (risk) of complications – in particular uncommon and serious ones" Policy 153

Crucially, very few policies (n=3, 3%) stated that clinicians should discuss the evidence or lack thereof associated with a procedure.

<u>*Alternative treatments*</u>

61 (67%) policies included guidance on discussing alternative treatments, the majority of which (n=45, 49%) used text extracted from the HSC document, which stated that patients need to "be informed about…alternatives, including no treatment"[12]. The remaining 16 policies used other synonymous language to communicate the same, for example:

"All alternative options for managing a condition must be described including refusing all treatment." Policy 3

7 (8%) policies provided further guidance on providing information about alternative treatments. Of these, three (3%) stated that patients should be given the comparative risks and benefits of alternative treatments, two (2%) outlined that "established" treatment options must be discussed with patients and two (2%) specified that treatments available elsewhere should also be discussed:

"Patients must be told what alternative treatments are available and what the risks and outcomes are for those treatments, both at this Trust and elsewhere" Policy 134

<u>*Surgeon's experience with the procedure*</u>

57 (63%) policies stated that clinicians should disclose their experience of performing the procedure. Of these, 44 (48%) stated that patients must be made aware of "the lack of experience of its (IP/D) use" – a statement identical to that used in the HSC document[12]. Of the remaining 13 (14%) policies, three (3%) stated that local (i.e. within trust) experiences should be discussed, five (5%) specifically stated that clinicians should discuss their personal experience with the procedure, and five (5%) stated both personal and local experience

For peer review only - http://bmjopen.bmj.com/site/about/guidelines.xhtml

should be discussed. Of the 91 policies, only four (4%) stipulated that training related to the procedure and/or any supervision (i.e. proctors), should be disclosed to patients:

> *"Valid consent must include an understanding that the procedure is new, that the practitioner is relatively inexperienced in the technique but (as appropriate) either well supervised or well trained" Policy 128*

Guidance on the use of written information

In total 67 (74%) policies included guidance regarding the use of written information for patients. Statements, such as the "proposal should include where relevant…a patient information leaflet" and "clinicians should consider providing written patient information", were common throughout many policies.

Although such statements suggested that clinicians should provide written patient information, it was unclear whether this was a mandatory stipulation. Only 30 included explicit instructions that written information must be provided to patients (e.g. "there must be written patient information" and "patients must be made aware in writing"). Ten (11%) also specified that written information would be subjected to an internal review before being approved:

> *"The [local NHS committee] will not consider the new interventional procedure unless an adequate draft PIL accompanies the application. The PIL will need to be approved by the divisional patient information readership panel." Policy 55*

Of the 67 policies that referenced written information, only 19 (21%) included guidance on the content that should be included. Of these, the specified content of the written information (and associated number of policies) were: evidence (n=1), surgeon's experience (n=3), special status/novelty (n=9), NICE status (n=4), alternative treatments (n=5) and potential risks and benefits (n=11) see appendix 2.

An example of a more comprehensive statement is provided below, which specified that written information must include the NICE status, potential risks and benefits, and alternative treatments to the IP/D. However, no singular policy specified that all the components listed above should be included in the written information.

For peer review only - http://bmjopen.bmj.com/site/about/guidelines.xhtml

“The information must include the safety and efficacy of the procedure, whether or not it has been favourably assessed by NICE and the risks, benefits and alternatives of the procedure.” Policy 102

Discussion

This is the first study to investigate guidance included in local written NHS policies on information provision and informed consent for patients being offered new IP/Ds being in NHS hospitals (which are not part of a research protocol). Interestingly, nearly a fifth (22/113) of all the policies did not include any statement on informed consent or lacked guidance on information to be provided to patients. Where guidance was provided, there was variation across policies’ regarding disclosure of the novel status of a procedure, potential risks and benefits (and uncertainties), alternative treatments and surgeon’s experience. We also found that whilst the majority of policies referred to patients receiving written information, this was often not a mandatory requirement and many policies did not state what information should be included. This highlights the considerable variation in NHS organisations’ guidance for information provision and informed consent for innovative IP/Ds.

Written patient information provided prior to surgery is crucial to improving patients’ understanding of surgery[27] and is recommend by RCS[2] as well as NICE.[28] The General Medical Council (GMC) and NICE further outline that patients should be given information in a format they prefer including written, audio, translated, through pictures or online.[17 28] Our findings demonstrate the absence of a mandatory requirement for specific written information when patients are being offered innovative procedures which could lead to patients lacking appropriate information and affect their ability to exercise informed consent. Regarding the content of the information provided, the GMC asserts that patients must be told the nature of their treatment option, including whether it is an innovative option.[17] Whilst our findings demonstrate that the majority of policies stated that the ‘special status’ of the procedure needs to be disclosed to patients, it is possible that this disclosure does not explicitly reveal the novelty of the procedure. Additionally, only three (3%) policies identified how long the procedures could be considered new or possessing a special status, resulting in ambiguity as to the length of time patients needed to be told about the procedure’s newness. The GMC

For peer review only - http://bmjopen.bmj.com/site/about/guidelines.xhtml

also states that potential risks, benefits and uncertainties should be discussed with patients.[17] This is also reiterated in the NICE guidelines for shared decision making, outlining that clinicians should openly discuss the risks, benefits and consequences of treatment choices with patients.[28] Although some of the policies examined highlighted the need to discuss risks, benefits and uncertainties with patients, only three (3%) stated that clinicians should discuss the evidence associated with the procedure. This was a notable omission across most of the policies given that discussion of evidence is an element of informed consent identified as important within multiple guidelines.[2 17 29]

Previous research has demonstrated that clinicians find discussing innovation with their patients challenging.[18] The absence of detail within local written NHS policies is likely to exacerbate this issue and highlights the need for more comprehensive and standardised guidelines. Such guidance could help support clinicians in openly discussing innovative surgery with patients. The basis for the amount of detail or depth of information to share with patients has been debated previously, with suggestions including: detail that a typical physician would share, detail that an average patient would understand, and bespoke detail for individual patients.[30] In the UK, the expectation is for clinicians to tailor the information to individual patients, taking into consideration the individual patient's values and desires.[31] Previous research has demonstrated that clinicians' opinions on what information is important and how much information to provide to patients can be varied, when offering innovative surgery,[32 33] a phenomenon that can lead to gaps in patient information provision.

It has been postulated that informed consent in reference to surgical innovation (within neurology) needs to include discussions on novelty, risk/benefit ratio, surgeon's experience, potential conflicts of interest, alternatives to the innovative procedure and knowledge or lack thereof of the long-term outcomes.[34] However, our findings highlight that the policies used across all clinical departments contain variable guidance across NHS organisations, this could lead to uncertainty on what exactly patients need to be told before, during and after undergoing innovative invasive procedures. While our study identified different types of information to share with patients, further research is needed to ascertain which types of information and what level of detail would be appropriate and helpful to patients when considering innovative procedures. Of particular interest would be the development of a core information set which is the minimum information to be given by a clinician in all

For peer review only - http://bmjopen.bmj.com/site/about/guidelines.xhtml

consultations for a particular operation,[35] and in this case within the context of innovative invasive procedures.[36]

A major strength of this study is that it has sought to systematically evaluate guidance on patient information and consent in local written NHS policies on introducing new IP/Ds in England and Wales. To date, very few studies have conducted research on informed consent in the context of surgical innovation, and to our knowledge, none have explored local written NHS policies. Our study highlights the absence of and the variability in guidance, therefore raising concerns about the information patients might receive. Adequate information provision to patients constitutes part of the governance for the introduction of new invasive procedures; this is needed not only to protect patients from ineffective and potentially harmful surgeries but also to uphold their autonomy in the decision-making for treatment which is a key element of biomedical ethics.[37] A limitation of this study is that we cannot ascertain from the current findings if and how informed consent practices are implemented by clinicians , whether they know about these policies and whether other regulatory processes within NHS organisations play a role in patient consent. Additionally, while our findings provide an overview of governance systems used across NHS organisations, it is important to note that a few of the NHS organisations approached did not respond to our request for policy documents (n=9, 5%). Of those that responded, there is the potential that not all relevant documents (such as explanatory appendices) were shared with us. These documents may have provided further information on informed consent that could have implications on our findings. Based on our findings, we postulate that comprehensive and standardised guidelines on information provision and consent for innovative IP/Ds could facilitate discussions and provide clarity to clinicians regarding the types of information and the level of detail to disclose to patients. This could enhance patients' understanding and facilitate their decision-making based on robust informed consent.

Author contributions

JMB developed the idea for the study with support from SC, HSR, DE and RH. The methodology was developed by JMB, RH, SC, HSR, DE, KA, BGM and NSB. HSR, SC, JMB, RH, JZ, and JM developed the data extraction form. HSR extracted free text from policy

For peer review only - http://bmjopen.bmj.com/site/about/guidelines.xhtml

documents, JZ analysed the data with support from JM, HSR and DE. The manuscript was written by CO and JZ, with critical revisions made by the wider introduce study team (JMB, DE, SC, HSR, SP, JM, JM, CO, JZ, KA, BGM, and NSB). The NIHR Bristol & Weston Biomedical Research Centre Surgical Innovation theme is directed by JMB (theme lead) and RH (deputy theme lead). The corresponding author attests that all listed authors meet authorship criteria and that no others meeting the criteria have been omitted.

Acknowledgement

We would like to thank Harry Robertson (HR) for his contribution to the reviewing and data extraction of 20% of the policy documents for the study.

Footnotes

The lead author (CAO) affirms that the manuscript is an honest, accurate, and transparent account of the study being reported; that no important aspects of the study have been omitted, and that any discrepancies from the study as planned have been explained.

I, the Submitting Author (CAO) has the right to grant and does grant on behalf of all authors of the Work (as defined in the author licence), an exclusive licence and/or a non-exclusive licence for contributions from authors who are: i) UK Crown employees; ii) where BMJ has agreed a CC-BY licence shall apply, and/or iii) in accordance with the terms applicable for US Federal Government officers or employees acting as part of their official duties; on a worldwide, perpetual, irrevocable, royalty-free basis to BMJ Publishing Group Ltd ("BMJ") its licensees. The Submitting Author (CAO) accepts and understands that any supply made under these terms is made by BMJ to the Submitting Author unless you are acting as an employee on behalf of your employer or a postgraduate student of an affiliated institution which is paying any applicable article publishing charge ("APC") for Open Access articles. Where the Submitting Author wishes to make the Work available on an Open Access basis (and intends to pay the relevant APC), the terms of reuse of such Open Access shall be governed by a Creative Commons licence – details of these licences and which licence will apply to this Work are set out in our licence referred to above.

The last author (JMB) has the right to grant on behalf of all authors and does grant on behalf of all authors, a worldwide licence to the Publishers and its licensees in perpetuity, in all

For peer review only

forms, formats and media (whether known now or created in the future), to i) publish, reproduce, distribute, display and store the Contribution, ii) translate the Contribution into other languages, create adaptations, reprints, include within collections and create summaries, extracts and/or, abstracts of the Contribution, iii) create any other derivative work(s) based on the Contribution, iv) to exploit all subsidiary rights in the Contribution, v) the inclusion of electronic links from the Contribution to third party material where-ever it may be located; and, vi) licence any third party to do any or all of the above.

No competing interests: All authors have completed the ICMJE uniform disclosure form at http://www.icmje.org/disclosure-of-interest/ and declare: no support from any organisation for the submitted work; no financial relationships with any organisations that might have an interest in the submitted work in the previous three years; no other relationships or activities that could appear to have influenced the submitted work.

Funding: This study was supported by the NIHR Biomedical Research Centre at University Hospitals Bristol & Weston NHS Foundation Trust and the University of Bristol, the MRC ConDuCT-II (Collaboration and innovation for Difficult and Complex randomised controlled Trials In Invasive procedures) Hub for Trials Methodology Research (MR/K025643/1) and a NIHR senior investigator award (NF-SI-0514-10114). The views expressed in this publication are those of the author(s) and not necessarily those of the NHS, the National Institute for Health Research or the Department of Health and Social Care.

Ethics approval: Not required.

Data sharing: The raw data will not be shared in order to protect the anonymity of the participating organisations.

Dissemination to participants and related patient and public communities: Results from the study will be disseminated through presentations to the public and specialists audiences through conferences and workshops.

Legend: figure 1- flowchart of policy documents reviewed

References

1. Maynard A, Bloor K. The NHS at 60: the next 60 years. *J R Soc Med* 2008;101(7):345-49. doi: 10.1258/jrsm.2008.nh8006

For peer review only - http://bmjopen.bmj.com/site/about/guidelines.xhtml

2. Royal College of Surgeons. Surgical innovation, new techniques and technologies 2019.
3. Ljungqvist O, Scott M, Fearon KC. Enhanced Recovery After Surgery: A Review. *JAMA Surgery* 2017;152(3):292-98. doi: 10.1001/jamasurg.2016.4952
4. James G. Report of the Independent Inquiry into the Issues raised by Paterson. London: Government of the United Kingdom, 2020.
5. Cumberlege J. First Do No Harm. The report of the Independent Medicines and Medical Devices Safety Review. London: Government of the United Kingdom, 2020.
6. Kerrison S, McNally N, Pollock AM. United Kingdom research governance strategy. *BMJ* 2003;327(7414):553-56. doi: 10.1136/bmj.327.7414.553
7. Lyratzopoulos G, Patrick H, Campbell B. Registers needed for new interventional procedures. *The Lancet* 2008;371(9626):1734-36. doi: 10.1016/S0140-6736(08)60742-4
8. Blencowe N, Chana P, Whistance R, et al. Outcome reporting in neoadjuvant surgical trials: a systematic review of the literature and proposals for new standards. *JNCI: Journal of the National Cancer Institute* 2014;106(9)
9. Blencowe NS, McNair AG, Davis CR, et al. Standards of outcome reporting in surgical oncology: a case study in esophageal cancer. *Annals of surgical oncology* 2012;19(13):4012-18.
10. Currie A, Brigic A, Blencowe N, et al. Systematic review of surgical innovation reporting in laparoendoscopic colonic polyp resection. *Journal of British Surgery* 2015;102(2):e108-e16.
11. Whistance R, Forsythe R, McNair A, et al. A systematic review of outcome reporting in colorectal cancer surgery. *Colorectal Disease* 2013;15(10):e548-e60.
12. Department of Health. Health Service Circular (HSC2003/011) – The Interventional Procedures Programme 2003 [Available from: https://webarchive.nationalarchives.gov.uk/20120503190439/http://www.dh.gov.uk/prod_consum_dh/groups/dh_digitalassets/@dh/@en/documents/digitalasset/dh_4064925.pdf accessed 12/02/2018.
13. National Institute for Health and Care Excellence. Interventional procedures programme manual Process and methods: NICE, 2016.
14. Cousins S, Richards H, Zahra J, et al. Introduction and adoption of innovative invasive procedures and devices in the NHS: an in-depth analysis of written policies and qualitative interviews (the introduce study protocol). *BMJ open* 2019;9(8):e029963.
15. Entwistle VA, Carter SM, Cribb A, et al. Supporting patient autonomy: the importance of clinician-patient relationships. *Journal of general internal medicine* 2010;25(7):741-45.
16. Chan SW, Tulloch E, Cooper ES, et al. Montgomery and informed consent: where are we now? 2017;357:j2224.
17. General Medical Council. Guidance on professional standards and ethics for doctors

Decision making and consent: GMC; 2020 [Available from: https://www.gmc-uk.org/-/media/documents/gmc-guidance-for-doctors---decision-making-and-consent-english_pdf-84191055.pdf?la=en&hash=BE327A1C584627D12BC51F66E790443F0E0651DA accessed 01/03/2021 2021.
18. Zahra J, Paramasivan S, Blencowe NS, et al. Discussing surgical innovation with patients: a qualitative study of surgeons' and governance representatives' views. *BMJ open* 2020;10(11):e035251.

For peer review only - http://bmjopen.bmj.com/site/about/guidelines.xhtml

19. Hardicre J. Valid informed consent in research: An introduction. *British Journal of Nursing* 2014;23(11):564-67.
20. Royal College of Surgeons. Learning from invited reviews. 2019
21. Procedures–Surgical NI, Safety A. ASERNIP S. 2007
22. Freedman DB. Introducing new interventional procedures within a District General Hospital, linking with the process for implementing NICE guidance. *Clinical Governance: An International Journal* 2006;11(3):180-86. doi: 10.1108/14777270610683100
23. Cousins S, Blencowe NS, Blazeby JM. What is an invasive procedure? A definition to inform study design, evidence synthesis and research tracking. *BMJ open* 2019;9(7):e028576.
24. Elo S, Kyngäs H. The qualitative content analysis process. *Journal of advanced nursing* 2008;62(1):107-15.
25. Lucas PJ, Baird J, Arai L, et al. Worked examples of alternative methods for the synthesis of qualitative and quantitative research in systematic reviews. *BMC medical research methodology* 2007;7(1):1-7.
26. Thomas J, Harden A. Methods for the thematic synthesis of qualitative research in systematic reviews. *BMC medical research methodology* 2008;8(1):1-10.
27. Sustersic M, Gauchet A, Foote A, et al. How best to use and evaluate Patient Information Leaflets given during a consultation: a systematic review of literature reviews. 2017;20(4):531-42. doi: 10.1111/hex.12487
28. NICE. Shared decision making: NICE, 2021.
29. National Institute for Clinical Excellence. Consent – procedures for which the benefits and risks are uncertain. In: Health Do, ed. London: NICE, 2003.
30. Sarin D, Agarwal BB, Rao B. Ethics for surgeons: The role of trainees, surgical innovations and the informed consent. *ETHICS* 2007;20(3):185.
31. McKinnon C, Loughran D, Finn R, et al. Surgical consent practice in the UK following the Montgomery ruling: a national cross-sectional questionnaire study. *International Journal of Surgery* 2018;55:66-72.
32. Reitsma AM, Moreno JD. Ethical regulations for innovative surgery: the last frontier? *Journal of the American College of Surgeons* 2002;194(6):792-801. doi: https://doi.org/10.1016/S1072-7515(02)01153-5
33. Lee Char SJ, Hills NK, Lo B, et al. Informed consent for innovative surgery: a survey of patients and surgeons. *Surgery* 2013;153(4):473-80. doi: 10.1016/j.surg.2012.08.068 [published Online First: 2012/12/04]
34. Ammirati M, Rosenfeld J, Hulsbergen A, et al. Ethical aspects of innovation in neurosurgery. *Ethics in neurosurgical practice* 2020:205.
35. Blazeby J, Macefield R, Blencowe N, et al. Core information set for oesophageal cancer surgery. *Journal of British Surgery* 2015;102(8):936-43.
36. Main BG, McNair AG, Huxtable R, et al. Core information sets for informed consent to surgical interventions: baseline information of importance to patients and clinicians. *BMC medical ethics* 2017;18(1):1-9.
37. Beauchamp TL, Childress JF. Principles of biomedical ethics: Oxford University Press, USA 2001.

For peer review only - http://bmjopen.bmj.com/site/about/guidelines.xhtml

For peer review only

For peer review only - http://bmjopen.bmj.com/site/about/guidelines.xhtml

Figure 1 Flow chart of policy documents reviewed

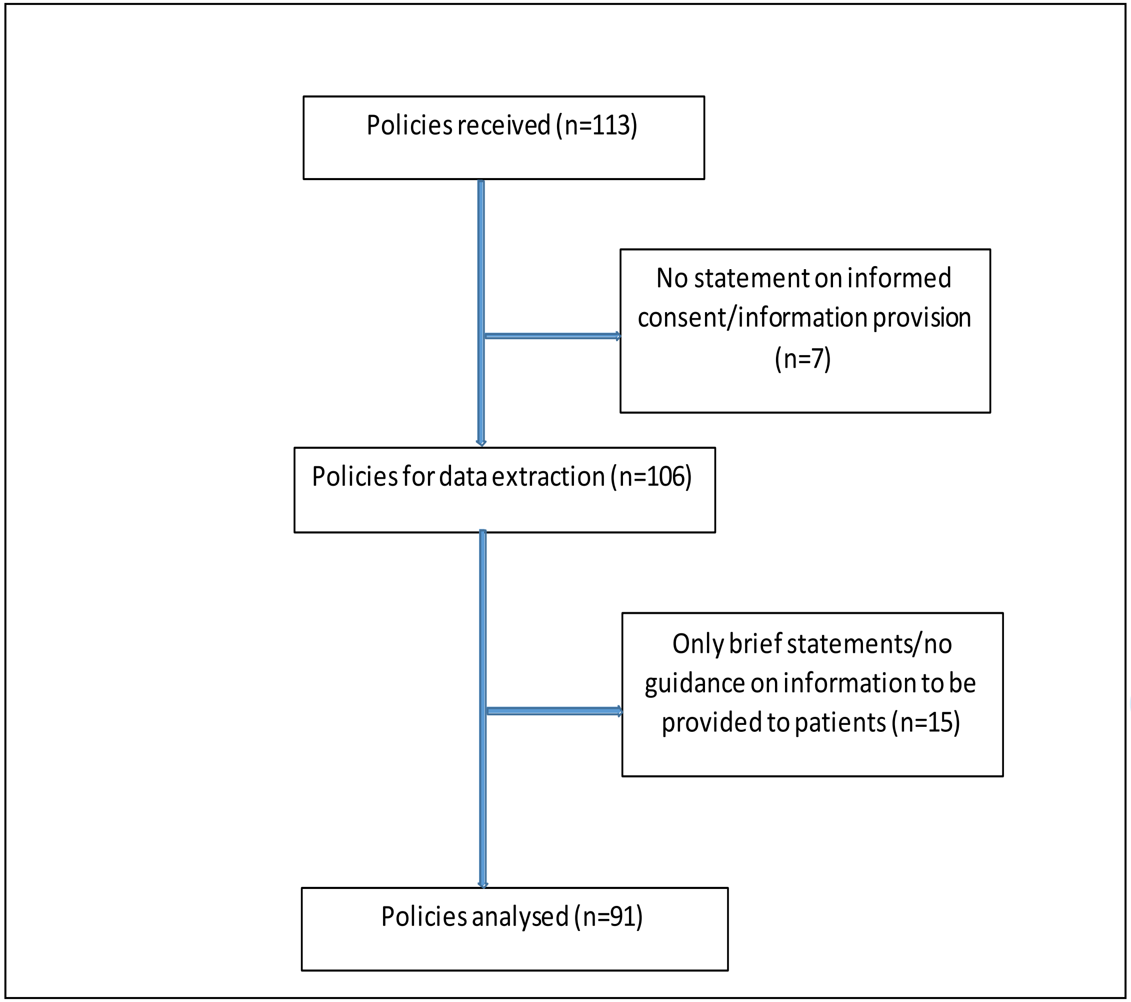

For peer review only - http://bmjopen.bmj.com/site/about/guidelines.xhtml

For peer review only

Supplementary file

Appendix 1 information to tell patients about new IP/Ds

Policy number	Newness or special status (N=73, 80%)	Referenced PIL or written information (N=68, 75%)	Alternative treatments (N=61, 67%)	Uncertainty regarding procedure (N=57, 63%)	Surgeon's experience (N=57, 63%)	Mandated PIL (N=30, 33%)	Total
14	Yes	Yes	Yes	Yes	Yes	Yes	6/6
19	Yes	Yes	Yes	Yes	Yes	Yes	6/6
22	Yes	Yes	Yes	Yes	Yes	Yes	6/6
41	Yes	Yes	Yes	Yes	Yes	Yes	6/6
49	Yes	Yes	Yes	Yes	Yes	Yes	6/6
51	Yes	Yes	Yes	Yes	Yes	Yes	6/6
55	Yes	Yes	Yes	Yes	Yes	Yes	6/6
58	Yes	Yes	Yes	Yes	Yes	Yes	6/6
81	Yes	Yes	Yes	Yes	Yes	Yes	6/6
88	Yes	Yes	Yes	Yes	Yes	Yes	6/6
95	Yes	Yes	Yes	Yes	Yes	Yes	6/6
146	Yes	Yes	Yes	Yes	Yes	Yes	6/6
91	Yes	Yes	Yes	Yes	Yes	Yes	6/6
13	Yes	Yes	Yes	Yes	No	Yes	5/6
21	Yes	Yes	No	Yes	Yes	Yes	5/6
26	Yes	Yes	Yes	Yes	Yes	No	5/6
38	Yes	Yes	Yes	Yes	Yes	No	5/6
39	Yes	Yes	Yes	No	Yes	Yes	5/6
50	Yes	Yes	Yes	Yes	No	Yes	5/6
54	Yes	Yes	Yes	Yes	Yes	No	5/6
56	Yes	Yes	Yes	Yes	Yes	No	5/6
65	Yes	Yes	No	Yes	Yes	Yes	5/6
76	Yes	Yes	Yes	Yes	Yes	No	5/6
105	Yes	Yes	Yes	Yes	Yes	No	5/6

110	Yes	Yes	Yes	Yes	Yes	No	5/6
118	Yes	Yes	Yes	Yes	Yes	No	5/6
122	Yes	Yes	Yes	Yes	Yes	No	5/6
126	Yes	Yes	Yes	Yes	Yes	No	5/6
132	Yes	Yes	Yes	Yes	Yes	No	5/6
150	Yes	Yes	Yes	Yes	Yes	No	5/6
153	Yes	Yes	Yes	Yes	Yes	No	5/6
12	Yes	Yes	Yes	yes	Yes	No	5/6
3	No	Yes	Yes	Yes	No	Yes	4/6
4	Yes	Yes	Yes	Yes	No	No	4/6
7	Yes	Yes	No	No	Yes	Yes	4/6
10	Yes	No	Yes	Yes	Yes	No	4/6
18	Yes	No	Yes	Yes	Yes	No	4/6
32	Yes	No	Yes	Yes	Yes	No	4/6
42	Yes	No	Yes	Yes	Yes	No	4/6
43	Yes	Yes	No	Yes	Yes	No	4/6
48	Yes	No	Yes	Yes	Yes	No	4/6
57	Yes	Yes	Yes	No	No	Yes	4/6
63	Yes	No	Yes	Yes	Yes	No	4/6
71	Yes	No	Yes	Yes	Yes	No	4/6
82	Yes	No	Yes	Yes	Yes	No	4/6
84	Yes	Yes	Yes	Yes	No	No	4/6
92	Yes	Yes	Yes	Yes	No	No	4/6
104	Yes	No	Yes	Yes	Yes	No	4/6
107	Yes	No	Yes	Yes	Yes	No	4/6
113	Yes	No	Yes	Yes	Yes	No	4/6
125	Yes	No	Yes	Yes	Yes	No	4/6
127	Yes	No	Yes	Yes	Yes	No	4/6
128	Yes	Yes	No	Yes	Yes	No	4/6
138	Yes	Yes	Yes	Yes	No	No	4/6

2	No	Yes	No	Yes	No	Yes	3/6
15	Yes	Yes	No	No	Yes	No	3/6
25	Yes	Yes	Yes	No	No	No	3/6
33	Yes	Yes	Yes	No	No	No	3/6
37	Yes	Yes	No	No	Yes	No	3/6
53	Yes	No	Yes	Yes	No	No	3/6
83	Yes	No	Yes	Yes	No	No	3/6
100	Yes	Yes	No	No	No	Yes	3/6
121	Yes	Yes	No	No	Yes	No	3/6
123	Yes	No	Yes	Yes	No	No	3/6
124	Yes	No	Yes	Yes	No	No	3/6
130	Yes	Yes	No	No	Yes	No	3/6
137	Yes	Yes	No	No	No	Yes	3/6
148	No	Yes	Yes	No	Yes	No	3/6
154	No	Yes	No	No	Yes	Yes	3/6
156	Yes	Yes	No	No	No	Yes	3/6
1	Yes	No	No	No	Yes	No	2/6
23	No	Yes	No	No	No	Yes	2/6
30	No	Yes	No	No	No	Yes	2/6
36	Yes	No	Yes	No	No	No	2/6
52	No	Yes	No	No	No	Yes	2/6
59	Yes	No	No	No	Yes	No	2/6
60	Yes	Yes	No	No	No	No	2/6
64	No	Yes	No	No	Yes	No	2/6
70	Yes	No	No	No	Yes	No	2/6
80	Yes	No	No	Yes	No	No	2/6
94	No	No	Yes	Yes	No	No	2/6
96	No	Yes	Yes	No	No	No	2/6
102	No	Yes	Yes	No	No	No	2/6
131	Yes	Yes	No	No	No	No	2/6

134	No	No	Yes	No	Yes	No	2/6
147	No	Yes	No	No	No	Yes	2/6
34	No	Yes	No	No	No	No	1/6
61	No	Yes	No	No	No	No	1/6
68	No	Yes	No	No	No	No	1/6
109	No	Yes	No	No	No	No	1/6
139	No	Yes	No	No	No	No	1/6

Appendix 2. Recommendation for information to be included in written patient information sheets

Policy Number	cc	Alternative Treatments (N=5, 26%)	NICE Status (N=4, 21%)	Special status/newness (N=9, 47%)	Surgeon's Experience (N=3, 16%)	Evidence (N=1, 5%)	Total
52	Yes	Yes	Yes	No	No	No	3/6
55	No	No	No	Yes	Yes	Yes	3/6
102	Yes	Yes	Yes	No	No	No	3/6
3	Yes	Yes	No	No	No	No	2/6
12	No	No	Yes	Yes	No	No	2/6
41	Yes	No	No	Yes	No	No	2/6
49	Yes	No	No	Yes	No	No	2/6
51	No	No	No	Yes	Yes	No	2/6
88	No	No	No	Yes	Yes	No	2/6
92	Yes	Yes	No	No	No	No	2/6
137	Yes	No	No	Yes	No	No	2/6
13	No	No	No	Yes	No	No	1/6
25	No	No	No	Yes	No	No	1/6
30	No	No	Yes	No	No	No	1/6
50	Yes	No	No	No	No	No	1/6
65	No	Yes	No	No	No	No	1/6

109	Yes	No	No	No	No	No	1/6
147	Yes	No	No	No	No	No	1/6
156	Yes	No	No	No	No	No	1/6

For peer review only

STROBE Statement—checklist of items that should be included in reports of observational studies

	Item No	Recommendation	Page No
**Title and abstract**	1	(*a*) Indicate the study's design with a commonly used term in the title or the abstract	1
		(*b*) Provide in the abstract an informative and balanced summary of what was done and what was found	2,3
**Introduction**			
Background/rationale	2	Explain the scientific background and rationale for the investigation being reported	4,5
Objectives	3	State specific objectives, including any prespecified hypotheses	5
**Methods**			
Study design	4	Present key elements of study design early in the paper	5
Setting	5	Describe the setting, locations, and relevant dates, including periods of recruitment, exposure, follow-up, and data collection	5
Participants	6	(*a*) *Cohort study*—Give the eligibility criteria, and the sources and methods of selection of participants. Describe methods of follow-up<br>*Case-control study*—Give the eligibility criteria, and the sources and methods of case ascertainment and control selection. Give the rationale for the choice of cases and controls<br>*Cross-sectional study*—Give the eligibility criteria, and the sources and methods of selection of participants	5
		(*b*) *Cohort study*—For matched studies, give matching criteria and number of exposed and unexposed<br>*Case-control study*—For matched studies, give matching criteria and the number of controls per case	
Variables	7	Clearly define all outcomes, exposures, predictors, potential confounders, and effect modifiers. Give diagnostic criteria, if applicable	Not applicable
Data sources/ measurement	8*	For each variable of interest, give sources of data and details of methods of assessment (measurement). Describe comparability of assessment methods if there is more than one group	*Supplementary file 1- Appendices*
Bias	9	Describe any efforts to address potential sources of bias	6
Study size	10	Explain how the study size was arrived at	5
Quantitative variables	11	Explain how quantitative variables were handled in the analyses. If applicable, describe which groupings were chosen and why	Not applicable
Statistical methods	12	(*a*) Describe all statistical methods, including those used to control for confounding	Not applicable
		(*b*) Describe any methods used to examine subgroups and interactions	Not applicable
		(*c*) Explain how missing data were addressed	

For peer review only - http://bmjopen.bmj.com/site/about/guidelines.xhtml
For peer review only

(*d*) *Cohort study*—If applicable, explain how loss to follow-up was addressed<br>*Case-control study*—If applicable, explain how matching of cases and controls was addressed<br>*Cross-sectional study*—If applicable, describe analytical methods taking account of sampling strategy	6
(*e*) Describe any sensitivity analyses	

Continued on next page

For peer review only

**Results**			
Participants	13*	(a) Report numbers of individuals at each stage of study—eg numbers potentially eligible, examined for eligibility, confirmed eligible, included in the study, completing follow-up, and analysed	5,7
		(b) Give reasons for non-participation at each stage	5.7
		(c) Consider use of a flow diagram	7- figure 1- flowchart of policy documents reviewed
Descriptive data	14*	(a) Give characteristics of study participants (eg demographic, clinical, social) and information on exposures and potential confounders	Not applicable
		(b) Indicate number of participants with missing data for each variable of interest	5,7
		(c) *Cohort study*—Summarise follow-up time (eg, average and total amount)	Not applicable
Outcome data	15*	*Cohort study*—Report numbers of outcome events or summary measures over time	Not applicable
		Case-control study—Report numbers in each exposure category, or summary measures of exposure	Not applicable
		Cross-sectional study—Report numbers of outcome events or summary measures	7-11
Main results	16	(*a*) Give unadjusted estimates and, if applicable, confounder-adjusted estimates and their precision (eg, 95% confidence interval). Make clear which confounders were adjusted for and why they were included	Not applicable
		(*b*) Report category boundaries when continuous variables were categorized	Not applicable
		(*c*) If relevant, consider translating estimates of relative risk into absolute risk for a meaningful time period	Not applicable
Other analyses	17	Report other analyses done—eg analyses of subgroups and interactions, and sensitivity analyses	Not applicable
**Discussion**			
Key results	18	Summarise key results with reference to study objectives	11
Limitations	19	Discuss limitations of the study, taking into account sources of potential bias or imprecision. Discuss both direction and magnitude of any potential bias	13
Interpretation	20	Give a cautious overall interpretation of results considering objectives, limitations, multiplicity of analyses, results from similar studies, and other relevant evidence	11-13
Generalisability	21	Discuss the generalisability (external validity) of the study results	11-13
**Other information**			
Funding	22	Give the source of funding and the role of the funders for the present study and, if applicable, for the original study on which the present article is based	14,15

*Give information separately for cases and controls in case-control studies and, if applicable, for exposed and unexposed groups in cohort and cross-sectional studies.

For peer review only

For peer review only - http://bmjopen.bmj.com/site/about/guidelines.xhtml

Note: An Explanation and Elaboration article discusses each checklist item and gives methodological background and published examples of transparent reporting. The STROBE checklist is best used in conjunction with this article (freely available on the Web sites of PLoS Medicine at http://www.plosmedicine.org/, Annals of Internal Medicine at http://www.annals.org/, and Epidemiology at http://www.epidem.com/). Information on the STROBE Initiative is available at www.strobe-statement.org.

For peer review only

For peer review only - http://bmjopen.bmj.com/site/about/guidelines.xhtml

BMJ Open

A qualitative documentary analysis of guidance on information provision and consent for the introduction of innovative invasive procedures including surgeries within NHS organisation policies in England and Wales.

Journal:	*BMJ Open*
Manuscript ID	bmjopen-2021-059228.R1
Article Type:	Original research
Date Submitted by the Author:	05-May-2022
Complete List of Authors:	Ochieng, Cynthia; University of Bristol, Population Health Sciences<br>Richards, Hollie; University of Bristol, Population Health Sciences<br>Zahra, Jesmond; University of Bristol, Population Health Sciences<br>Cousins, Sian; University of Bristol, Population Health Sciences<br>Elliott, Daisy; University of Bristol, Population Health Sciences<br>Wilson, Nicholas; University of Bristol, Population Health Sciences<br>Paramasivan, Sangeetha ; University of Bristol, Population Health Sciences<br>Avery, Kerry; University of Bristol, Population Health Sciences<br>Mathews, Johnny; University of Bristol, Population Health Sciences<br>Main, Barry; University of Bristol, Population Health Sciences; University Hospitals Bristol NHS Foundation Trust<br>Hinchliffe, Robert; University of Bristol, Population Health Sciences; North Bristol NHS Trust<br>Blencowe, Natalie; University of Bristol, Population Health Sciences; University Hospitals Bristol NHS Foundation Trust<br>Blazeby, Jane; University of Bristol, Population Health Sciences; University Hospitals Bristol NHS Foundation Trust
**Primary Subject Heading**:	Surgery
Secondary Subject Heading:	Qualitative research
Keywords:	Health policy < HEALTH SERVICES ADMINISTRATION & MANAGEMENT, Clinical governance < HEALTH SERVICES ADMINISTRATION & MANAGEMENT, ETHICS (see Medical Ethics), Protocols & guidelines < HEALTH SERVICES ADMINISTRATION & MANAGEMENT, SURGERY

SCHOLARONE™
Manuscripts

I, the Submitting Author has the right to grant and does grant on behalf of all authors of the Work (as defined in the below author licence), an exclusive licence and/or a non-exclusive licence for contributions from authors who are: i) UK Crown employees; ii) where BMJ has agreed a CC-BY licence shall apply, and/or iii) in accordance with the terms applicable for US Federal Government officers or employees acting as part of their official duties; on a worldwide, perpetual, irrevocable, royalty-free basis to BMJ Publishing Group Ltd ("BMJ") its licensees and where the relevant Journal is co-owned by BMJ to the co-owners of the Journal, to publish the Work in this journal and any other BMJ products and to exploit all rights, as set out in our licence.

The Submitting Author accepts and understands that any supply made under these terms is made by BMJ to the Submitting Author unless you are acting as an employee on behalf of your employer or a postgraduate student of an affiliated institution which is paying any applicable article publishing charge ("APC") for Open Access articles. Where the Submitting Author wishes to make the Work available on an Open Access basis (and intends to pay the relevant APC), the terms of reuse of such Open Access shall be governed by a Creative Commons licence – details of these licences and which Creative Commons licence will apply to this Work are set out in our licence referred to above.

Other than as permitted in any relevant BMJ Author's Self Archiving Policies, I confirm this Work has not been accepted for publication elsewhere, is not being considered for publication elsewhere and does not duplicate material already published. I confirm all authors consent to publication of this Work and authorise the granting of this licence.

For peer review only

A qualitative documentary analysis of guidance on information provision and consent for the introduction of innovative invasive procedures including surgeries within NHS organisation policies in England and Wales.

Submission category: Original research

Authors: Cynthia A Ochieng (0000-0002-5574-6059), senior research associate, c.ochieng@bristol.ac.uk[1], Hollie Richards (0000-0001-9181-7005), senior research associate, hollie.richards@bristol.ac.uk[1], Jesmond Zahra (0000-0002-7947-2216), senior research associate, jesmond.zahra@defra.gov.uk[1] , Sian Cousins (0000-0003-0088-841X), research fellow, sian.cousins@bristol.ac.uk[1], Daisy Elliott (0000-0001-8143-9549), research fellow, daisy.elliott@bristol.ac.uk[1], Nicholas Wilson, senior research associate, nicholas.wilson@bristol.ac.uk[1], Sangeetha Paramasivan (0000-0001-7329-9574), research fellow, sangeetha.paramasivan@bristol.ac.uk[2], Kerry NL Avery (0000-0001-5477-2418), senior lecturer, kerry.avery@bristol.ac.uk[1], Johnny Mathews, PhD fellow, johnny.mathews@bristol.ac.uk[1], Barry G Main (0000-0003-0622-805X), consultant senior lecturer, b.g.main@bristol.ac.uk[1,3], Robert Hinchliffe (0000-0002-6370-0800), clinical professor of vascular surgery, robert.hinchliffe@bristol.ac.uk[1,4], Natalie S Blencowe (0000-0002-6111-2175), associate professor in surgery, natalie.blencowe@bristol.ac.uk[1,3], Jane M Blazeby (0000-0002-3354-3330), professor of surgery, j.m.blazeby@bristol.ac.uk[1,3],

[1] National Institute for Health Research Bristol and Weston Biomedical Research Centre, Surgical Innovation Theme, Centre for Surgical Research, Population Health Sciences, Bristol Medical School, University of Bristol, 39 Whatley Road, Bristol, BS8 2PS

[2]Population Health Sciences, Bristol Medical School, University of Bristol, 39 Whatley Road, Bristol, BS8 2PS

[3]University Hospitals Bristol NHS Foundation Trust, Bristol, UK. BS2 8HW

[4]North Bristol NHS Trust, Bristol, United Kingdom

Correspondence to: Cynthia A Ochieng c.ochieng@bristol.ac.uk

Word count: 4621 (excluding title page, abstract, tables and references)

For peer review only

Abstract

Objective- To review guidance, included in written local NHS organisation policies, on information provision and consent for the introduction of new invasive procedures-including surgeries and devices (IP/Ds).

Design- A qualitative documentary analysis of data on patient information provision and consent extracted from policies for the introduction of IP/Ds from NHS organisations in England and Wales.

Setting- NHS trusts in England and health boards in Wales, United Kingdom.

Participants- Between December 2017 and July 2018 150 acute trusts in England and seven health boards in Wales were approached for their policies for the introduction of new IP/Ds. 123 policies were received, 11 did not fit the inclusion criteria and a further policy was included from a trust website resulting in 113 policies included for review.

Results- From the 113 policies, 22 did not include any statements on informed consent/information provision or lacked guidance on the information to be provided to patients and were hence excluded. Consequently, 91 written local NHS policies were included in the final dataset. Within the guidance obtained, variation existed on disclosure of the procedure's novelty, potential risks, benefits, uncertainties, alternative treatments, and surgeon's experience. Few policies stated that clinicians should discuss the existing evidence associated with a procedure. Additionally, whilst the majority of policies referred to patients needing written information, this was often not mandated and few policies specified the information to be included.

Conclusions- Nearly a fifth of all the policies lacked guidance on information to be provided to patients. There was variability in the policy documents regarding what patients should be told about innovative procedures. Further research is needed to ascertain the information and level of detail appropriate for patients when considering innovative procedures. A core information set including patients' and clinicians' views is required to address variability around information provision/ consent for innovative procedures.

For peer review only - http://bmjopen.bmj.com/site/about/guidelines.xhtml

Strengths and limitations of this study

- This is the first study to systematically review guidance within NHS policies on patient information provision and consent when introducing new invasive procedures and devices in England and Wales.
- The study explores not just the types of information included in the policies but also the level of detail.
- The study cannot ascertain if and how informed consent practices are implemented by clinicians and/or whether other regulatory processes play a role in patient consent.

For peer review only

For peer review only - http://bmjopen.bmj.com/site/about/guidelines.xhtml

Introduction

A singular definition of surgical innovation is challenging as it can be contextualised to different aspects of a procedure.[1] This may include an entirely novel or modified anatomical approach, differences in the use of devices and technology or changes to a patient group.[2] The degree of modification from standard procedures that constitutes an innovation is also often debated with implications on the governance required for the procedure[2]. Innovation in surgery has transformed how clinical care is delivered; new surgical techniques and technologies have resulted in improved outcomes, shorter recovery times and fewer adverse events following surgery.[3-5] National guidance for good surgical practice advices clinicians intending to introduce innovative procedures and/or devices (IP/Ds) to contact the Interventional procedures programme at the National Institute for Health and Care Excellence (NICE) to find out the novelty status of the procedure or to register it, and follow local NHS policies for approval if not being introduced as an NHS Ethics committee research programme.[6 7] The guidance also states that clinicians should obtain appropriate training in the procedure and participate in on-going training to develop competence as well as support the training of other clinicians,[6] the guidance however does not specify if the surgeons should report on their experience. Additionally, the guidance states that clinicians should contribute to the evaluation of the IP/Ds by reviewing progress and auditing outcomes and ensure devices are certified and comply with European standards and that patients are informed of the newness of the procedure as well as associated alternatives. [6]

Nonetheless, as documented in recent reports, surgical innovations are sometimes introduced into clinical practice without sufficient evaluation and pose unknown risks to patients.[8 9] Introducing new invasive procedures via research pathways helps ensure clinicians comply with legal, ethical and scientific standards.[10] Despite increasing demand for evidence-based practice, new surgical procedures are commonly delivered prior to appropriate evaluation and without research ethics approval.[11-15] Working in conjunction with the National Institute for Health and Care Excellence (NICE), the UK Department of Health and Social Care (DHSC) published guidelines for managing the introduction of new invasive procedures within the NHS.[16 17] These are relevant to NHS local 'new procedures committees/clinical effectiveness committees'.[18] These guidelines included sections on information provision and obtaining consent from patients particularly for procedures

For peer review only - http://bmjopen.bmj.com/site/about/guidelines.xhtml

introduced outside the remit of research protocols; these were distributed to NHS chief executives via the Health Service Circular (HSC) in 2003.[16]

Patient autonomy is considered a cornerstone of modern healthcare provision,[19] philosophically it is argued that respecting personal autonomy stems from acknowledging that each person has inherent worth and the ability to choose their own destiny.[20] Beauchamp and Childress [20] further explain that for one to exercise personal autonomy, they need to be intentional, done with understanding and be free from controlling influences of others. Respecting this autonomy hence promotes a person's right to hold views and make their own choices as well as supporting their capacity to make these decisions.[20] Clinicians must work in partnership with patients and take their views, goals and circumstances into consideration when establishing best treatment.[21 22] Informed consent can be seen as a process that fosters patient-centred decision-making by respecting their right to self-determination.[23] In the context of surgical innovation, in terms of ethics, informed consent is complicated by trying to communicate challenging aspects like the inherent uncertainties within the innovation, surgeon's lack of experience, lack of robust evidence for the procedure and cost implications.[24] There is a scarcity of empirical research exploring informed consent in the context of surgical innovation, consequently, little is known about how innovative surgeries are, or should be, discussed with patients[25]. When innovations are introduced as part of a research study, ethical review processes help ensure that patient information is appropriate and includes detail of the potential risks and benefits.[26] However, when procedures are introduced outside the remit of research studies these safeguards regarding patient consent for innovative surgeries are not always adhered to.[27]

While guidelines on consent practices for new technologies have recently been published,[4] there persists a paucity of research investigating how these are implemented in clinical practice. The Royal College of Surgeons (RCS) guidance state that as part of the consent process for treatment, patients need to be told of their diagnosis and prognosis, options for treatment including no treatment, the purpose, expected benefits and risks of the procedure, likelihood of success and potential further treatment as well as the clinicians that will be involved in the procedure.[28] The General Medical Council (GMC) guidance also add that patients should be told of the uncertainties with the diagnosis and prognosis as well as those associated with the treatment options.[29] The GMC also state that patients need to be told if

For peer review only - http://bmjopen.bmj.com/site/about/guidelines.xhtml

the treatment is innovative.[29] Both the RCS and GMC guidance state that the patient should be informed by a clinician who is trained and qualified to provide the treatment, and the RCS advises that clinicians should tell patients their experience with the procedure.[28-30] According to the 2003 HSC recommendations, new invasive procedures which have not been evaluated or recommended by NICE should only be granted approval if special consent measures are put in place.[16] The GMC has produced guidance stating that patients should be given information in their preferred format including written, pictures, audio, or other media[29] and the RCS state that where possible written information about diagnosis and treatment should be provided to patients.[6] The consent is expected to be documented in written form in the patients notes as well as through written consent forms in the case of invasive procedures.[6 29] This guidance, however, is not specific for innovative procedures being conducted exclusive of a research setting.

Most individual NHS organisations have policies in place regarding the introduction of new IP/Ds,[18 31 32] however, it is unknown what guidance these policies provide about information provision to patients undergoing innovative procedures. This review aimed to investigate information provision and consent measures for the introduction of new invasive procedures and devices described in policy documents within NHS organisations in England (trusts) and Wales (health boards).

Methods

Study design

A comprehensive qualitative documentary review of guidance on information provision and consent within NHS organisations' policies for the introduction of IP/Ds was undertaken. An IP/D was defined as any procedure where access is gained via an incision, natural orifice or percutaneous puncture or involving devices used inside the body[33]. The findings reported here form part of the INTRODUCE Study.[18]

Data collection

For peer review only - http://bmjopen.bmj.com/site/about/guidelines.xhtml

The first step in the qualitative documentary analysis as described by May [34] is conceptualising the documents appropriate for the study. In this research, written NHS policies produced by individual hospitals for the oversight of new and modified invasive procedures were the documents of interest. Whilst it is known such documents frequently exist there have to our knowledge not been previously studied or collated. Whilst NHS organisations were asked for their policies and guidance documents for innovative, new and modified procedures, we did not give them a definition of what these involved because of the lack of an agreed national definition. Between December 2017 and July 2018 all acute trusts in England (n=150) and health boards in Wales (n=7) were contacted (via email and telephone calls) and requested for their policies relating to the introduction of an innovative IP/D.[18] Of these, 147 responded and 127 stated that they had the requested policies. Of the 127 trusts and health boards, four did not share their policies with the research team. Consequently, 123 policies were received of which 11 were excluded from the review for: being device management policies (n=6), application forms only (n=3), committee terms of reference (n=1), and local safety standards for invasive procedures (n=1). Further detail on the eligibility criteria have been published in the main protocol.[18] In addition, one policy was obtained online from the trust website, resulting in 113 policies available for review.

Data extraction and analysis

The emphasis of the qualitative documentary analysis was initially to discover and subsequently describe[35] the contents of the policies. The next step in the qualitative documentary analysis involved iterative reading of the policies[36] by HSR. This can be described as the initial stage of the interpretation and is aimed at sub-dividing the document into different broad areas of focus.[37 38] For this study there were varied areas of focus all relating to innovative procedures, this paper reports on the findings of one of these areas- information provision and consent. All text relating to information provision and consent practice for new procedures and devices was extracted verbatim by HSR into a data extraction form. The data extraction form was iteratively developed through reviewing policy documents and previous literature[18] and stored on the online surveys portal (formerly Bristol online surveys). 20% of the policies were independently reviewed and data extraction was conducted by SC, JMB, JM, HR and JZ. Any disagreements among reviewers were discussed and resolved.

For peer review only - http://bmjopen.bmj.com/site/about/guidelines.xhtml

Extracted data relating to information provision and consent were then imported into NVivo (version 12) by JZ for review and analysis using methods derived from content analysis[39] and thematic synthesis.[40 41] This stage is often termed the reflective interpretation stage in qualitative documentary analysis[37 38]. This approach allowed for a thematic synthesis to explore the thematic patterns and depth of information while also permitting quantification of their frequencies through content analysis. [35] The extracted data were iteratively read to ensure familiarisation. Following this, coding categories were inductively formulated from reviewing the policies including the HSC document as in a grounded theory approach.[35] These coding categories were collated into a thematic framework. In total, five coding categories were included in the final thematic framework. To ensure this framework fully encapsulated the data, it was piloted on a sample of policies (15%), by two analysts (JZ and JM) independently before being applied to the full dataset. To promote rigor in the data analysis, frequent discussions were held with the team to discuss and refine the initial findings with reference to the raw data. Once the framework had been populated with all the data, it was possible to have a clear overview of each policy's data and analysis results on information provision and consent. This served as case description[37] from each policy, while also providing numerical frequencies of the policies that contained each theme in the framework. The completed framework also permitted the comparative analysis[37] between different policies highlighting similarities and differences among the policies.

Patient and public involvement

The review was conducted under the National Institute for Health Research (NIHR) Bristol & Weston Biomedical Research Centre (BRC) Surgical Innovation theme, which conducts research aimed at improving safety and transparent translation of innovative procedures and/or devices to clinical practice. The NIHR Bristol BRC has a dedicated patient and public involvement (PPI) group, where previous surgical patients contribute their views on different projects. In this study, PPI contributors included their views and experiences of the information they received or would have wanted to receive before and after having innovative surgeries. The PPI group were supportive of this study. Our PPI group agreed with the methods applied for the review of the policy documents as well as the resultant findings.

For peer review only - http://bmjopen.bmj.com/site/about/guidelines.xhtml

Results

Policies included

Of the 113 policies reviewed, seven did not include any statements on informed consent/information provision. Of the remaining 106, 15 provided only brief statements on informed consent (e.g. "consent must be obtained") and did not provide any guidance on the content of information to be provided to patients. This highlights that 19% (22/113) of all the policies did not include any statement on informed consent or lacked guidance on information to be provided to patients. Following discussions within the team, these 15 policies were excluded from further analysis. In total, text from 91 local NHS policies was included in the final dataset (see figure 1- flowchart of policy documents reviewed).

Findings

Five categories were included in the final thematic framework and grouped into two main themes: statements about the information clinicians should disclose about a new IP/D being delivered with local NHS policy approval, and guidance on the use of written information for this situation. The findings in each of the five categories from the analytic framework are described in detail below. Very few policies included guidance on all of the five categories identified (n = 14, see appendix 1).

Statements specifying what information clinicians should disclose prior to delivering an IP/D

"New/special" status of an IP/D

Of the final 91 policies reviewed, 73 (80%) policies stipulated that clinicians should discuss the novelty of a procedure with patients, however, the terminology used within policies varied. 48 (53%) stated the "special status" (n = 44) or "status" (n = 4) of a procedure should be disclosed. This terminology mirrored that used in the HSC document distributed in 2003[16]:

For peer review only - http://bmjopen.bmj.com/site/about/guidelines.xhtml

"All patients offered the procedure are made aware of the special status of the procedure"

Of the remaining policies, 20 (22%) used the term "new" and five (5%) included statements that used both "new" and "special status". Notably, only one policy stated clinicians should explain whether the procedure was "new to the trust" or "completely new":

"An explanation must be given to the patient as to whether the new technique / procedure is either new to the Trust but established elsewhere, or, is a completely new technique / procedure." Policy 81

Three (3%) policies provided guidance on how long clinicians should consider a procedure as new and introduce it to patients as such, however, guidance varied between policies. Specific recommendations were that, i) standard consent processes should be applied following a successful audit of the procedure, ii) clinicians should review their results and decide when a procedure is an established part of practice, and iii) procedures should be introduced as "new" to the first five patients.

Uncertainty, evidence, risks and benefits of a procedure

58 (64%) policies stated that clinicians should discuss uncertainties surrounding safety and efficacy, and/or risks and benefits. Of these, 40 (44%) included an extract from the HSC document[16]:

"Patients need to understand that the procedure's safety and efficacy is uncertain and be informed about the anticipated benefits and possible adverse effects of the procedure"

12 (13%) of the remaining policies simply stated that patients need to be "*informed"* that some uncertainty existed for a procedure.

Five policies provided more comprehensive guidelines for clinicians to follow when discussing uncertainty. For example, one policy stated that the frequency of procedure complications should also be disclosed:

"The most important consideration is that patients (or their parents or carers, when appropriate) should be informed and should understand the

For peer review only - http://bmjopen.bmj.com/site/about/guidelines.xhtml

risks when offered the procedure. This always means telling them the known risks, but in addition it may mean telling them that there is uncertainty about the frequency (risk) of complications – in particular uncommon and serious ones" Policy 153

Crucially, very few policies (n=3, 3%) stated that clinicians should discuss the evidence or lack thereof associated with a procedure.

Alternative treatments

61 (67%) policies included guidance on discussing alternative treatments, the majority of which (n=45, 49%) used text extracted from the HSC document, which stated that patients need to "be informed about…alternatives, including no treatment"[16]. The remaining 16 policies used other synonymous language to communicate the same, for example:

"All alternative options for managing a condition must be described including refusing all treatment." Policy 3

7 (8%) policies provided further guidance on providing information about alternative treatments. Of these, three (3%) stated that patients should be given the comparative risks and benefits of alternative treatments, two (2%) outlined that "established" treatment options must be discussed with patients and two (2%) specified that treatments available elsewhere should also be discussed:

"Patients must be told what alternative treatments are available and what the risks and outcomes are for those treatments, both at this Trust and elsewhere" Policy 134

Surgeon's experience with the procedure

57 (63%) policies stated that clinicians should disclose their experience of performing the procedure. Of these, 44 (48%) stated that patients must be made aware of "the lack of experience of its (IP/D) use" – a statement identical to that used in the HSC document[16]. Of the remaining 13 (14%) policies, three (3%) stated that local (i.e. within trust) experiences should be discussed, five (5%) specifically stated that clinicians should discuss their personal

For peer review only - http://bmjopen.bmj.com/site/about/guidelines.xhtml

experience with the procedure, and five (5%) stated both personal and local experience should be discussed. Of the 91 policies, only four (4%) stipulated that training related to the procedure and/or any supervision (i.e. proctors), should be disclosed to patients:

> *"Valid consent must include an understanding that the procedure is new, that the practitioner is relatively inexperienced in the technique but (as appropriate) either well supervised or well trained" Policy 128*

Guidance on the use of written information

In total 67 (74%) policies included guidance regarding the use of written information for patients. Statements, such as the "proposal should include where relevant…a patient information leaflet" and "clinicians should consider providing written patient information", were common throughout many policies.

Although such statements suggested that clinicians should provide written patient information, it was unclear whether this was a mandatory stipulation. Only 30 included explicit instructions that written information must be provided to patients (e.g. "there must be written patient information" and "patients must be made aware in writing"). Ten (11%) also specified that written information would be subjected to an internal review before being approved:

> *"The [local NHS committee] will not consider the new interventional procedure unless an adequate draft PIL accompanies the application. The PIL will need to be approved by the divisional patient information readership panel." Policy 55*

Of the 67 policies that referenced written information, only 19 (21%) included guidance on the content that should be included. Of these, the specified content of the written information (and associated number of policies) were: evidence (n=1), surgeon's experience (n=3), special status/novelty (n=9), NICE status (n=4), alternative treatments (n=5) and potential risks and benefits (n=11) see appendix 2.

An example of a more comprehensive statement is provided below, which specified that written information must include the NICE status, potential risks and benefits, and alternative

For peer review only - http://bmjopen.bmj.com/site/about/guidelines.xhtml

treatments to the IP/D. However, no singular policy specified that all the components listed above should be included in the written information.

"The information must include the safety and efficacy of the procedure, whether or not it has been favourably assessed by NICE and the risks, benefits and alternatives of the procedure." Policy 102

Discussion

This is the first study to investigate guidance included in local written NHS policies on information provision and informed consent for patients being offered new IP/Ds in NHS hospitals (which are not part of a research protocol). Interestingly, nearly a fifth (22/113) of all the policies did not include any statement on informed consent or lacked guidance on information to be provided to patients. Where guidance was provided, there was variation across policies' regarding disclosure of the novel status of a procedure, potential risks and benefits (and uncertainties), alternative treatments and surgeon's experience. We also found that whilst the majority of policies referred to patients receiving written information, this was often not a mandatory requirement and many policies did not state what information should be included. This highlights the considerable variation in NHS organisations' guidance for information provision and informed consent for innovative IP/Ds. Additionally, many policies had similar wording to the HSC guidance document[16] pointing to the intertextuality often found in bureaucratized documents where texts may appear similar and cross-referencing is not uncommon.[36] It can be postulated that many of the polices we received had been formulated based on the HSC guidance document,[16] any bid to update the policies may require the issuance of a similar guidance through the HSC to NHS organisations.

Written patient information provided prior to surgery is crucial to improving patients' understanding of surgery[42] and is recommended in guidance by RCS[4] as well as NICE.[43] Fundamentally, informed consent should be an exercise of patient autonomy by ensuring that patients understand the procedure and its implications as well as being free of any controlling influences.[20] The GMC and NICE further outline that patients should be given information in a format they prefer including written, audio, translated, through pictures or online.[22 43] Our findings demonstrate the absence of a mandatory requirement for specific written

For peer review only - http://bmjopen.bmj.com/site/about/guidelines.xhtml

information when patients are being offered innovative procedures which could lead to patients lacking appropriate information and affect their ability to exercise informed consent. The inability to exercise informed consent philosophically contravenes the autonomy of the patient and in so doing impacts on their power and positioning within the clinical decision-making process.[20] Documenting the consent in consent forms before a patient undergoes an invasive procedure is usual practice. The RCS cautions that the consent process should be more than just form filling, their guidance on good surgical practice highlight that it should be informed decision-making supported by clarity in explanation of the diagnosis, prognosis and available procedures.[6] Their guidance also states that the consent forms should be completed at the end of the discussion, and stored in the patient notes alongside a description there-in of the discussions had, and re-affirmed on the day of the procedure. [6] The RCS and GMC guidance, however, provide limited advice on informed consent for innovative procedures. This is the subject of on-going research at the Bristol BioMedical Research Centre.

Regarding the content of the information provided, the GMC asserts that patients must be told the nature of their treatment option, including whether it is an innovative option.[22] Whilst our findings demonstrate that the majority of policies stated that the 'special status' of the procedure needs to be disclosed to patients, it is possible that this disclosure does not explicitly reveal the novelty of the procedure. Additionally, only three (3%) policies identified how long the procedures could be considered new or possessing a special status, resulting in ambiguity as to the length of time patients needed to be told about the procedure's newness. Standardising the length of time for which IP/Ds should be considered innovative is unpragmatic because different procedures and devices require different times, data and length of experience to move from the novel status to standard care.[44] According to the IDEAL framework, surgical innovation can be divided into four main stages: innovation, development, exploration, assessment and long-term implementation and monitoring with each stage requiring its own unique time.[44] The point at which the IP/D is no longer an innovation is therefore more of a conceptual exercise at the long-term implementation stage of the innovation. At that point, the procedure is a part of routine practice and special consent relating to innovative procedures is no longer considered to be needed.[44] In view of these difficulties, it is considered that magnitude of risk and unknown risk is a more important way of defining 'innovation' or something that differs from routine practice.[1] The GMC also states

For peer review only - http://bmjopen.bmj.com/site/about/guidelines.xhtml

that potential risks, benefits and uncertainties should be discussed with patients.[22] This is also reiterated in the NICE guidelines for shared decision making, outlining that clinicians should openly discuss the risks, benefits and consequences of treatment choices with patients[43] as has debates in other clinical forums.[24] Although some of the policies examined highlighted the need to discuss risks, benefits and uncertainties with patients, only three (3%) stated that clinicians should discuss the evidence associated with the procedure. This was a notable omission across most of the policies given that discussion of evidence is an element of informed consent identified as important within multiple guidelines.[4 22 45]

Previous research has demonstrated that clinicians find discussing innovation with their patients challenging.[25] The absence of detail within local written NHS policies is likely to exacerbate this issue and highlights the need for more comprehensive and standardised guidelines. Such guidance could help support clinicians in openly discussing innovative surgery with patients. The basis for the amount of detail or depth of information to share with patients has been debated previously, with suggestions including: detail that a typical physician would share, detail that an average patient would understand, and bespoke detail for individual patients.[46] In the UK, the expectation is for clinicians to tailor the information to individual patients, taking into consideration the individual patient's values and desires.[47] Previous research has demonstrated that clinicians' opinions on what information is important and how much information to provide to patients can be varied, when offering innovative surgery,[48 49] a phenomenon that can lead to gaps in patient information provision.

It has been postulated that informed consent in reference to surgical innovation (within neurology) needs to include discussions on novelty, risk/benefit ratio, surgeon's experience, potential conflicts of interest, alternatives to the innovative procedure and knowledge or lack thereof of the long-term outcomes.[50] Whether clinicians are required to declare conflict of interest when offering patients innovative procedures is a subject of debate. Guidance from the GMC state that if relevant, clinicians should inform patients of any conflicts of interest that they or their organisation may have.[29] Whilst financial conflicts are expected to be made explicit, there is less guidance about declaring other conflicts of interest.[51] This is pertinent to innovation where personal prestige can be a relevant factor. The on-going research at the Bristol BioMedical Research Centre is exploring these issues with the aim of making recommendations to improve transparency. However, our findings highlight that the policies

For peer review only - http://bmjopen.bmj.com/site/about/guidelines.xhtml

used across all clinical departments contain variable guidance across NHS organisations, this could lead to uncertainty on what exactly patients need to be told before, during and after undergoing innovative invasive procedures. While our study identified different types of information to share with patients, further research is needed to ascertain which types of information and what level of detail would be appropriate and helpful to patients when considering innovative procedures. Of particular interest would be the development of a core information set which is the minimum information to be given by a clinician in all consultations for a particular operation,[52] and in this case within the context of innovative invasive procedures.[52 53]

A major strength of this study is that it has sought to systematically evaluate guidance on patient information and consent in local written NHS policies on introducing new IP/Ds in England and Wales. To date, very few studies have conducted research on informed consent in the context of surgical innovation, and to our knowledge, none have explored local written NHS policies. Our study highlights the absence of and the variability in guidance, therefore raising concerns about the information patients might receive. Adequate information provision to patients constitutes part of the governance for the introduction of new invasive procedures; this is needed not only to protect patients from ineffective and potentially harmful surgeries but also to uphold their autonomy in the decision-making for treatment which is a key element of biomedical ethics.[20] A limitation of this study is that we cannot ascertain from the current findings if and how informed consent practices are implemented by clinicians , whether they know about these policies and whether other regulatory processes within NHS organisations play a role in patient consent. Additionally, while our findings provide an overview of governance systems used across NHS organisations, it is important to note that a few of the NHS organisations approached did not respond to our request for policy documents (n=9, 5%). Of those that responded, there is the potential that not all relevant documents (such as explanatory appendices) were shared with us. These documents may have provided further information on informed consent that could have implications on our findings. Based on our findings, we postulate that comprehensive and standardised guidelines on information provision and consent for innovative IP/Ds could facilitate discussions and provide clarity to clinicians regarding the types of information and

For peer review only - http://bmjopen.bmj.com/site/about/guidelines.xhtml

the level of detail to disclose to patients. This could enhance patients' understanding and facilitate their decision-making based on robust informed consent.

Author contributions

JMB developed the idea for the study with support from SC, HSR, DE and RH. The methodology was developed by JMB, RH, SC, HSR, DE, KA, BGM and NSB. HSR, SC, JMB, RH, JZ, and JM developed the data extraction form. HSR extracted free text from policy documents, JZ analysed the data with support from JM, HSR and DE. The manuscript was written by CO and JZ, with critical revisions made by the wider introduce study team (JMB, DE, SC, HSR, SP, JM, CO, JZ, KA, BGM, NW and NSB). The NIHR Bristol & Weston Biomedical Research Centre Surgical Innovation theme is directed by JMB (theme lead) and RH (deputy theme lead). The corresponding author attests that all listed authors meet authorship criteria and that no others meeting the criteria have been omitted.

Acknowledgement

We would like to thank Harry Robertson (HR) for his contribution to the reviewing and data extraction of 20% of the policy documents for the study.

Footnotes

The lead author (CAO) affirms that the manuscript is an honest, accurate, and transparent account of the study being reported; that no important aspects of the study have been omitted, and that any discrepancies from the study as planned have been explained.

I, the Submitting Author (CAO) has the right to grant and does grant on behalf of all authors of the Work (as defined in the author licence), an exclusive licence and/or a non-exclusive licence for contributions from authors who are: i) UK Crown employees; ii) where BMJ has agreed a CC-BY licence shall apply, and/or iii) in accordance with the terms applicable for US Federal Government officers or employees acting as part of their official duties; on a worldwide, perpetual, irrevocable, royalty-free basis to BMJ Publishing Group Ltd ("BMJ") its licensees. The Submitting Author (CAO) accepts and understands that any supply made under these terms is made by BMJ to the Submitting Author unless you are acting as an employee on behalf of your employer or a postgraduate student of an affiliated institution which is

For peer review only - http://bmjopen.bmj.com/site/about/guidelines.xhtml

paying any applicable article publishing charge ("APC") for Open Access articles. Where the Submitting Author wishes to make the Work available on an Open Access basis (and intends to pay the relevant APC), the terms of reuse of such Open Access shall be governed by a Creative Commons licence – details of these licences and which licence will apply to this Work are set out in our licence referred to above.

The last author (JMB) has the right to grant on behalf of all authors and does grant on behalf of all authors, a worldwide licence to the Publishers and its licensees in perpetuity, in all forms, formats and media (whether known now or created in the future), to i) publish, reproduce, distribute, display and store the Contribution, ii) translate the Contribution into other languages, create adaptations, reprints, include within collections and create summaries, extracts and/or, abstracts of the Contribution, iii) create any other derivative work(s) based on the Contribution, iv) to exploit all subsidiary rights in the Contribution, v) the inclusion of electronic links from the Contribution to third party material where-ever it may be located; and, vi) licence any third party to do any or all of the above.

No competing interests: All authors have completed the ICMJE uniform disclosure form at http://www.icmje.org/disclosure-of-interest/ and declare: no support from any organisation for the submitted work; no financial relationships with any organisations that might have an interest in the submitted work in the previous three years; no other relationships or activities that could appear to have influenced the submitted work.

Funding: This study was supported by the NIHR Biomedical Research Centre at University Hospitals Bristol & Weston NHS Foundation Trust and the University of Bristol, the MRC ConDuCT-II (Collaboration and innovation for Difficult and Complex randomised controlled Trials In Invasive procedures) Hub for Trials Methodology Research (MR/K025643/1) and a NIHR senior investigator award (NF-SI-0514-10114). The views expressed in this publication are those of the author(s) and not necessarily those of the NHS, the National Institute for Health Research or the Department of Health and Social Care.

Ethics approval: Not required.

Data sharing: The raw data will not be shared in order to protect the anonymity of the participating organisations.

For peer review only

For peer review only - http://bmjopen.bmj.com/site/about/guidelines.xhtml

Dissemination to participants and related patient and public communities: Results from the study will be disseminated through presentations to the public and specialists audiences through conferences and workshops.

Legend: figure 1- flowchart of policy documents reviewed

References

1. Birchley G, Ives J, Huxtable R, et al. Conceptualising surgical innovation: an eliminativist proposal. *Health Care Analysis* 2020;28(1):73-97.
2. Hutchison K, Rogers W, Eyers A, et al. Getting clearer about surgical innovation. *Annals of surgery* 2015;262(6):949-54.
3. Maynard A, Bloor K. The NHS at 60: the next 60 years. *J R Soc Med* 2008;101(7):345-49. doi: 10.1258/jrsm.2008.nh8006
4. Royal College of Surgeons. Surgical innovation, new techniques and technologies 2019.
5. Ljungqvist O, Scott M, Fearon KC. Enhanced Recovery After Surgery: A Review. *JAMA Surgery* 2017;152(3):292-98. doi: 10.1001/jamasurg.2016.4952
6. Royal College of Surgeons. Good Surgical Practice. London: RCS, 2014.
7. Interventional procedures advisory committee. NICE interventional procedures guidance: NICE; [Available from: https://www.nice.org.uk/Get-Involved/Meetings-in-public/Interventional-Procedures-Advisory-Committee.
8. James G. Report of the Independent Inquiry into the Issues raised by Paterson. London: Government of the United Kingdom, 2020.
9. Cumberlege J. First Do No Harm. The report of the Independent Medicines and Medical Devices Safety Review. London: Government of the United Kingdom, 2020.
10. Kerrison S, McNally N, Pollock AM. United Kingdom research governance strategy. *BMJ* 2003;327(7414):553-56. doi: 10.1136/bmj.327.7414.553
11. Lyratzopoulos G, Patrick H, Campbell B. Registers needed for new interventional procedures. *The Lancet* 2008;371(9626):1734-36. doi: 10.1016/S0140-6736(08)60742-4
12. Blencowe N, Chana P, Whistance R, et al. Outcome reporting in neoadjuvant surgical trials: a systematic review of the literature and proposals for new standards. *JNCI: Journal of the National Cancer Institute* 2014;106(9)
13. Blencowe NS, McNair AG, Davis CR, et al. Standards of outcome reporting in surgical oncology: a case study in esophageal cancer. *Annals of surgical oncology* 2012;19(13):4012-18.
14. Currie A, Brigic A, Blencowe N, et al. Systematic review of surgical innovation reporting in laparoendoscopic colonic polyp resection. *Journal of British Surgery* 2015;102(2):e108-e16.
15. Whistance R, Forsythe R, McNair A, et al. A systematic review of outcome reporting in colorectal cancer surgery. *Colorectal Disease* 2013;15(10):e548-e60.
16. Department of Health. Health Service Circular (HSC2003/011) – The Interventional Procedures Programme 2003 [Available from: https://webarchive.nationalarchives.gov.uk/20120503190439/http://www.dh.gov.uk/prod_consum_dh/groups/dh_digitalassets/@dh/@en/documents/digitalasset/dh_4064925.pdf accessed 12/02/2018.

For peer review only - http://bmjopen.bmj.com/site/about/guidelines.xhtml

17. National Institute for Health and Care Excellence. Interventional procedures programme manual Process and methods: NICE, 2016.
18. Cousins S, Richards H, Zahra J, et al. Introduction and adoption of innovative invasive procedures and devices in the NHS: an in-depth analysis of written policies and qualitative interviews (the introduce study protocol). *BMJ open* 2019;9(8):e029963.
19. Entwistle VA, Carter SM, Cribb A, et al. Supporting patient autonomy: the importance of clinician-patient relationships. *Journal of general internal medicine* 2010;25(7):741-45.
20. Beauchamp TL, Childress JF. Principles of biomedical ethics: Oxford University Press, USA 2001.
21. Chan SW, Tulloch E, Cooper ES, et al. Montgomery and informed consent: where are we now? 2017;357:j2224.
22. General Medical Council. Guidance on professional standards and ethics for doctors
Decision making and consent: GMC; 2020 [Available from: https://www.gmc-uk.org/-/media/documents/gmc-guidance-for-doctors---decision-making-and-consent-english_pdf-84191055.pdf?la=en&hash=BE327A1C584627D12BC51F66E790443F0E0651DA accessed 01/03/2021 2021.
23. Bernat JL, Peterson LM. Patient-centered informed consent in surgical practice. *Archives of Surgery* 2006;141(1):86-92.
24. Angelos P. Surgical ethics and the challenge of surgical innovation. *The American Journal of Surgery* 2014;208(6):881-85.
25. Zahra J, Paramasivan S, Blencowe NS, et al. Discussing surgical innovation with patients: a qualitative study of surgeons' and governance representatives' views. *BMJ open* 2020;10(11):e035251.
26. Hardicre J. Valid informed consent in research: An introduction. *British Journal of Nursing* 2014;23(11):564-67.
27. Royal College of Surgeons. Learning from invited reviews. 2019
28. Royal College of Surgeons. Consent: Supported decision-making. A guide to good practice. London: RCS,, 2018.
29. General Medical Council. Decision making and consent content. London: GMC, 2020.
30. Royal College of Surgeons. Surgical innovation, new techniques and technologies London: RCS, 2019.
31. Procedures–Surgical NI, Safety A. ASERNIP S. 2007
32. Freedman DB. Introducing new interventional procedures within a District General Hospital, linking with the process for implementing NICE guidance. *Clinical Governance: An International Journal* 2006;11(3):180-86. doi: 10.1108/14777270610683100
33. Cousins S, Blencowe NS, Blazeby JM. What is an invasive procedure? A definition to inform study design, evidence synthesis and research tracking. *BMJ open* 2019;9(7):e028576.
34. May T. Social research: Issues, methods and research: McGraw-Hill Education (UK) 2011.
35. Altheide D, Coyle M, DeVriese K, et al. Emergent qualitative document analysis. *Handbook of emergent methods* 2008:127-51.
36. Shaw S, Elston J, Abbott S. Comparative analysis of health policy implementation: the use of documentary analysis. *Policy Studies* 2004;25(4):259-66.
37. Bohnsack R. Documentary method. *The SAGE handbook of qualitative data analysis* 2014:217-33.

For peer review only - http://bmjopen.bmj.com/site/about/guidelines.xhtml

38. Reischl J, Plotz T. Documentary method. *analyzing group interactions, M Huber and DE Froehlich Eds London & New York: Routledge Taylor & Francis Group* 2020
39. Elo S, Kyngäs H. The qualitative content analysis process. *Journal of advanced nursing* 2008;62(1):107-15.
40. Lucas PJ, Baird J, Arai L, et al. Worked examples of alternative methods for the synthesis of qualitative and quantitative research in systematic reviews. *BMC medical research methodology* 2007;7(1):1-7.
41. Thomas J, Harden A. Methods for the thematic synthesis of qualitative research in systematic reviews. *BMC medical research methodology* 2008;8(1):1-10.
42. Sustersic M, Gauchet A, Foote A, et al. How best to use and evaluate Patient Information Leaflets given during a consultation: a systematic review of literature reviews. 2017;20(4):531-42. doi: 10.1111/hex.12487
43. NICE. Shared decision making: NICE, 2021.
44. Barkun JS, Aronson JK, Feldman LS, et al. Evaluation and stages of surgical innovations. *The Lancet* 2009;374(9695):1089-96.
45. National Institute for Clinical Excellence. Consent – procedures for which the benefits and risks are uncertain. In: Health Do, ed. London: NICE, 2003.
46. Sarin D, Agarwal BB, Rao B. Ethics for surgeons: The role of trainees, surgical innovations and the informed consent. *ETHICS* 2007;20(3):185.
47. McKinnon C, Loughran D, Finn R, et al. Surgical consent practice in the UK following the Montgomery ruling: a national cross-sectional questionnaire study. *International Journal of Surgery* 2018;55:66-72.
48. Reitsma AM, Moreno JD. Ethical regulations for innovative surgery: the last frontier? *Journal of the American College of Surgeons* 2002;194(6):792-801. doi: https://doi.org/10.1016/S1072-7515(02)01153-5
49. Lee Char SJ, Hills NK, Lo B, et al. Informed consent for innovative surgery: a survey of patients and surgeons. *Surgery* 2013;153(4):473-80. doi: 10.1016/j.surg.2012.08.068 [published Online First: 2012/12/04]
50. Ammirati M, Rosenfeld J, Hulsbergen A, et al. Ethical aspects of innovation in neurosurgery. *Ethics in neurosurgical practice* 2020:205.
51. Barbour V, Clark J, Peiperl L, et al. Making sense of non-financial competing interests. *PLoS Medicine* 2008;5(9):1299-302.
52. Blazeby J, Macefield R, Blencowe N, et al. Core information set for oesophageal cancer surgery. *Journal of British Surgery* 2015;102(8):936-43.
53. Main BG, McNair AG, Huxtable R, et al. Core information sets for informed consent to surgical interventions: baseline information of importance to patients and clinicians. *BMC medical ethics* 2017;18(1):1-9.

For peer review only - http://bmjopen.bmj.com/site/about/guidelines.xhtml

Figure 1 Flow chart of policy documents reviewed

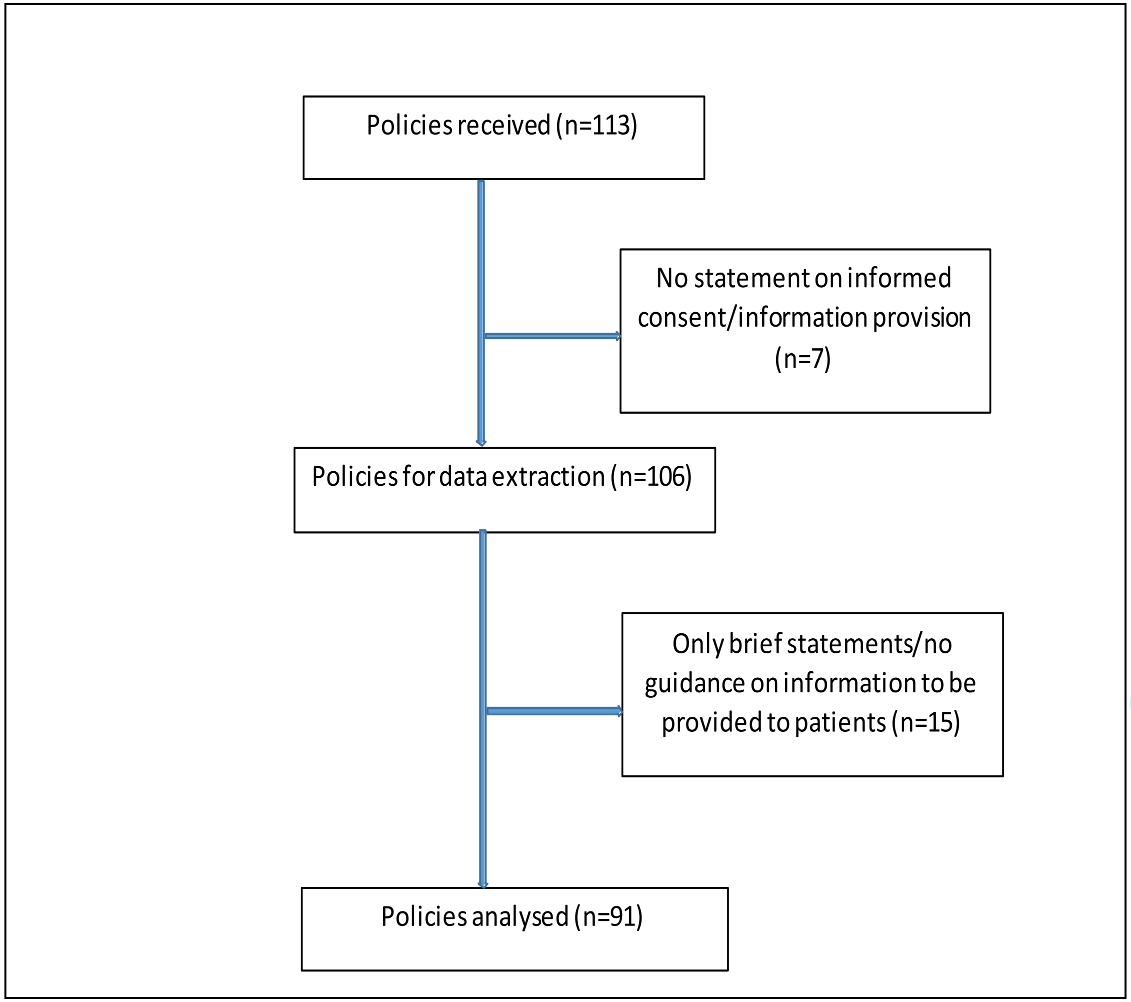

only

For peer review only

Supplementary file

Appendix 1 information to tell patients about new IP/Ds

Policy number	Newness or special status (N=73, 80%)	Referenced PIL or written information (N=68, 75%)	Alternative treatments (N=61, 67%)	Uncertainty regarding procedure (N=57, 63%)	Surgeon's experience (N=57, 63%)	Mandated PIL (N=30, 33%)	Total
14	Yes	Yes	Yes	Yes	Yes	Yes	6/6
19	Yes	Yes	Yes	Yes	Yes	Yes	6/6
22	Yes	Yes	Yes	Yes	Yes	Yes	6/6
41	Yes	Yes	Yes	Yes	Yes	Yes	6/6
49	Yes	Yes	Yes	Yes	Yes	Yes	6/6
51	Yes	Yes	Yes	Yes	Yes	Yes	6/6
55	Yes	Yes	Yes	Yes	Yes	Yes	6/6
58	Yes	Yes	Yes	Yes	Yes	Yes	6/6
81	Yes	Yes	Yes	Yes	Yes	Yes	6/6
88	Yes	Yes	Yes	Yes	Yes	Yes	6/6
95	Yes	Yes	Yes	Yes	Yes	Yes	6/6
146	Yes	Yes	Yes	Yes	Yes	Yes	6/6
91	Yes	Yes	Yes	Yes	Yes	Yes	6/6
13	Yes	Yes	Yes	Yes	No	Yes	5/6
21	Yes	Yes	No	Yes	Yes	Yes	5/6
26	Yes	Yes	Yes	Yes	Yes	No	5/6
38	Yes	Yes	Yes	Yes	Yes	No	5/6
39	Yes	Yes	Yes	No	Yes	Yes	5/6
50	Yes	Yes	Yes	Yes	No	Yes	5/6
54	Yes	Yes	Yes	Yes	Yes	No	5/6
56	Yes	Yes	Yes	Yes	Yes	No	5/6
65	Yes	Yes	No	Yes	Yes	Yes	5/6
76	Yes	Yes	Yes	Yes	Yes	No	5/6
105	Yes	Yes	Yes	Yes	Yes	No	5/6

110	Yes	Yes	Yes	Yes	Yes	No	5/6
118	Yes	Yes	Yes	Yes	Yes	No	5/6
122	Yes	Yes	Yes	Yes	Yes	No	5/6
126	Yes	Yes	Yes	Yes	Yes	No	5/6
132	Yes	Yes	Yes	Yes	Yes	No	5/6
150	Yes	Yes	Yes	Yes	Yes	No	5/6
153	Yes	Yes	Yes	Yes	Yes	No	5/6
12	Yes	Yes	Yes	yes	Yes	No	5/6
3	No	Yes	Yes	Yes	No	Yes	4/6
4	Yes	Yes	Yes	Yes	No	No	4/6
7	Yes	Yes	No	No	Yes	Yes	4/6
10	Yes	No	Yes	Yes	Yes	No	4/6
18	Yes	No	Yes	Yes	Yes	No	4/6
32	Yes	No	Yes	Yes	Yes	No	4/6
42	Yes	No	Yes	Yes	Yes	No	4/6
43	Yes	Yes	No	Yes	Yes	No	4/6
48	Yes	No	Yes	Yes	Yes	No	4/6
57	Yes	Yes	Yes	No	No	Yes	4/6
63	Yes	No	Yes	Yes	Yes	No	4/6
71	Yes	No	Yes	Yes	Yes	No	4/6
82	Yes	No	Yes	Yes	Yes	No	4/6
84	Yes	Yes	Yes	Yes	No	No	4/6
92	Yes	Yes	Yes	Yes	No	No	4/6
104	Yes	No	Yes	Yes	Yes	No	4/6
107	Yes	No	Yes	Yes	Yes	No	4/6
113	Yes	No	Yes	Yes	Yes	No	4/6
125	Yes	No	Yes	Yes	Yes	No	4/6
127	Yes	No	Yes	Yes	Yes	No	4/6
128	Yes	Yes	No	Yes	Yes	No	4/6
138	Yes	Yes	Yes	Yes	No	No	4/6

2	No	Yes	No	Yes	No	Yes	3/6
15	Yes	Yes	No	No	Yes	No	3/6
25	Yes	Yes	Yes	No	No	No	3/6
33	Yes	Yes	Yes	No	No	No	3/6
37	Yes	Yes	No	No	Yes	No	3/6
53	Yes	No	Yes	Yes	No	No	3/6
83	Yes	No	Yes	Yes	No	No	3/6
100	Yes	Yes	No	No	No	Yes	3/6
121	Yes	Yes	No	No	Yes	No	3/6
123	Yes	No	Yes	Yes	No	No	3/6
124	Yes	No	Yes	Yes	No	No	3/6
130	Yes	Yes	No	No	Yes	No	3/6
137	Yes	Yes	No	No	No	Yes	3/6
148	No	Yes	Yes	No	Yes	No	3/6
154	No	Yes	No	No	Yes	Yes	3/6
156	Yes	Yes	No	No	No	Yes	3/6
1	Yes	No	No	No	Yes	No	2/6
23	No	Yes	No	No	No	Yes	2/6
30	No	Yes	No	No	No	Yes	2/6
36	Yes	No	Yes	No	No	No	2/6
52	No	Yes	No	No	No	Yes	2/6
59	Yes	No	No	No	Yes	No	2/6
60	Yes	Yes	No	No	No	No	2/6
64	No	Yes	No	No	Yes	No	2/6
70	Yes	No	No	No	Yes	No	2/6
80	Yes	No	No	Yes	No	No	2/6
94	No	No	Yes	Yes	No	No	2/6
96	No	Yes	Yes	No	No	No	2/6
102	No	Yes	Yes	No	No	No	2/6
131	Yes	Yes	No	No	No	No	2/6

134	No	No	Yes	No	Yes	No	2/6
147	No	Yes	No	No	No	Yes	2/6
34	No	Yes	No	No	No	No	1/6
61	No	Yes	No	No	No	No	1/6
68	No	Yes	No	No	No	No	1/6
109	No	Yes	No	No	No	No	1/6
139	No	Yes	No	No	No	No	1/6

Appendix 2. Recommendation for information to be included in written patient information sheets

Policy Number	cc	Alternative Treatments (N=5, 26%)	NICE Status (N=4, 21%)	Special status/newness (N=9, 47%)	Surgeon's Experience (N=3, 16%)	Evidence (N=1, 5%)	Total
52	Yes	Yes	Yes	No	No	No	3/6
55	No	No	No	Yes	Yes	Yes	3/6
102	Yes	Yes	Yes	No	No	No	3/6
3	Yes	Yes	No	No	No	No	2/6
12	No	No	Yes	Yes	No	No	2/6
41	Yes	No	No	Yes	No	No	2/6
49	Yes	No	No	Yes	No	No	2/6
51	No	No	No	Yes	Yes	No	2/6
88	No	No	No	Yes	Yes	No	2/6
92	Yes	Yes	No	No	No	No	2/6
137	Yes	No	No	Yes	No	No	2/6
13	No	No	No	Yes	No	No	1/6
25	No	No	No	Yes	No	No	1/6
30	No	No	Yes	No	No	No	1/6
50	Yes	No	No	No	No	No	1/6
65	No	Yes	No	No	No	No	1/6

109	Yes	No	No	No	No	No	1/6
147	Yes	No	No	No	No	No	1/6
156	Yes	No	No	No	No	No	1/6

For peer review only

Standards for Reporting Qualitative Research (SRQR)*

http://www.equator-network.org/reporting-guidelines/srqr/

Title and abstract

	Page/line no(s).
**Title** - Concise description of the nature and topic of the study Identifying the study as qualitative or indicating the approach (e.g., ethnography, grounded theory) or data collection methods (e.g., interview, focus group) is recommended	1
**Abstract** - Summary of key elements of the study using the abstract format of the intended publication; typically includes background, purpose, methods, results, and conclusions	2

Introduction

**Problem formulation** - Description and significance of the problem/phenomenon studied; review of relevant theory and empirical work; problem statement	4-6
**Purpose or research question** - Purpose of the study and specific objectives or questions	6

Methods

**Qualitative approach and research paradigm** - Qualitative approach (e.g., ethnography, grounded theory, case study, phenomenology, narrative research) and guiding theory if appropriate; identifying the research paradigm (e.g., postpositivist, constructivist/ interpretivist) is also recommended; rationale**	6
**Researcher characteristics and reflexivity** - Researchers' characteristics that may influence the research, including personal attributes, qualifications/experience, relationship with participants, assumptions, and/or presuppositions; potential or actual interaction between researchers' characteristics and the research questions, approach, methods, results, and/or transferability	1
**Context** - Setting/site and salient contextual factors; rationale**	7
**Sampling strategy** - How and why research participants, documents, or events were selected; criteria for deciding when no further sampling was necessary (e.g., sampling saturation); rationale**	7
**Ethical issues pertaining to human subjects** - Documentation of approval by an appropriate ethics review board and participant consent, or explanation for lack thereof; other confidentiality and data security issues	N/A
**Data collection methods** - Types of data collected; details of data collection procedures including (as appropriate) start and stop dates of data collection and analysis, iterative process, triangulation of sources/methods, and modification of procedures in response to evolving study findings; rationale**	7

For peer review only

For peer review only - http://bmjopen.bmj.com/site/about/guidelines.xhtml

**Data collection instruments and technologies** - Description of instruments (e.g., interview guides, questionnaires) and devices (e.g., audio recorders) used for data collection; if/how the instrument(s) changed over the course of the study	7
**Units of study** - Number and relevant characteristics of participants, documents, or events included in the study; level of participation (could be reported in results)	9
**Data processing** - Methods for processing data prior to and during analysis, including transcription, data entry, data management and security, verification of data integrity, data coding, and anonymization/de-identification of excerpts	7-8
**Data analysis** - Process by which inferences, themes, etc., were identified and developed, including the researchers involved in data analysis; usually references a specific paradigm or approach; rationale**	8
**Techniques to enhance trustworthiness** - Techniques to enhance trustworthiness and credibility of data analysis (e.g., member checking, audit trail, triangulation); rationale**	8

Results/findings

**Synthesis and interpretation** - Main findings (e.g., interpretations, inferences, and themes); might include development of a theory or model, or integration with prior research or theory	9-13
**Links to empirical data** - Evidence (e.g., quotes, field notes, text excerpts, photographs) to substantiate analytic findings	10-13

Discussion

**Integration with prior work, implications, transferability, and contribution(s) to the field -** Short summary of main findings; explanation of how findings and conclusions connect to, support, elaborate on, or challenge conclusions of earlier scholarship; discussion of scope of application/generalizability; identification of unique contribution(s) to scholarship in a discipline or field	13-17
**Limitations** - Trustworthiness and limitations of findings	16-17

Other

**Conflicts of interest** - Potential sources of influence or perceived influence on study conduct and conclusions; how these were managed	18
**Funding** - Sources of funding and other support; role of funders in data collection, interpretation, and reporting	18

*The authors created the SRQR by searching the literature to identify guidelines, reporting standards, and critical appraisal criteria for qualitative research; reviewing the reference lists of retrieved sources; and contacting experts to gain feedback. The SRQR aims to improve the transparency of all aspects of qualitative research by providing clear standards for reporting qualitative research.

For peer review only

For peer review only - http://bmjopen.bmj.com/site/about/guidelines.xhtml

**The rationale should briefly discuss the justification for choosing that theory, approach, method, or technique rather than other options available, the assumptions and limitations implicit in those choices, and how those choices influence study conclusions and transferability. As appropriate, the rationale for several items might be discussed together.

Reference:

O'Brien BC, Harris IB, Beckman TJ, Reed DA, Cook DA. **Standards for reporting qualitative research: a synthesis of recommendations.** *Academic Medicine*, Vol. 89, No. 9 / Sept 2014 DOI: 10.1097/ACM.0000000000000388

For peer review only

For peer review only - http://bmjopen.bmj.com/site/about/guidelines.xhtml
